# Linezolid‐mediated Prevention of Fibroblast Activation and Tissue Fibrosis via Mitochondrial Translation Inhibition

**DOI:** 10.1002/art.43440

**Published:** 2026-02-13

**Authors:** Xuezhi Hong, Yanhua Xiao, Haodong Qi, Shihao Zhu, Tim Filla, Andrea‐Hermina Györfi, Yi‐Nan Li, Meilin Xu, Langxian Zhi, Thuong Trinh‐Minh, Clara Dees, Georg Schett, Jörg H. W. Distler, Alexandru‐Emil Matei

**Affiliations:** ^1^ Department of Rheumatology University Hospital Düsseldorf, Medical Faculty of Heinrich Heine University Düsseldorf Germany; ^2^ Hiller Research Center, University Hospital Düsseldorf, Medical Faculty of Heinrich Heine University Düsseldorf Germany; ^3^ Department of Internal Medicine 3, Rheumatology and Clinical Immunology Friedrich‐Alexander‐University Erlangen‐Nürnberg and University Hospital Erlangen Erlangen Germany; ^4^ Deutsches Zentrum Immuntherapie, University Hospital Erlangen Erlangen Germany; ^5^ Fraunhofer Institute for Translational Medicine and Pharmacology and Fraunhofer Cluster of Excellence for Immune‐Mediated Diseases Frankfurt am Main Germany

## Abstract

**Objective:**

Beyond its role as a ribosome‐targeting antibiotic, linezolid was recently shown to modulate immune responses by inhibiting mitochondrial translation. Because mitochondrial dysfunction is implicated in various fibrotic diseases, including systemic sclerosis (SSc), this study aimed to evaluate the antifibrotic potential of linezolid and delineate its underlying mechanisms in SSc.

**Methods:**

The effects of linezolid on fibrotic tissue remodeling were assessed using multiple experimental systems: human dermal fibroblasts, human macrophages, three‐dimensional SSc skin equivalents (SScSE), the murine model of sclerodermatous chronic graft‐versus‐host disease (sclGvHD) and precision‐cut skin slices (PCSS) obtained from patients with SSc, RNA sequencing, immunofluorescence, Western blot, and histology. Mitochondrial function was evaluated using Seahorse assays alongside mitochondrial protein synthesis assessments.

**Results:**

Linezolid inhibited TGFβ‐induced fibroblast activation in cultured human fibroblasts, SScSE, sclGvHD mice, and SSc‐PCSS, as demonstrated by reversal of profibrotic gene expression programs, downregulation of TGFβ, WNT, and JAK‐STAT signaling, and reductions in αSMA expression or stress fiber formation, which led to reduced collagen deposition and ameliorated skin or lung fibrosis in vivo. Mechanistically, linezolid induced metabolic alterations by inhibiting mitochondrial translation in fibroblasts to hamper the oxidative phosphorylation, reduce the NAD^+^/NADH ratio, and down‐regulate glycolysis, an essential metabolic pathway for fibroblast activation.

**Conclusion:**

This study provides the first evidence that inhibiting mitochondrial translation with linezolid ameliorates fibrotic tissue remodeling. Because linezolid is already clinically approved as a reserve antibiotic, these findings hold translational promise and support the use of linezolid as a novel treatment for fibrotic disorders after further validation.

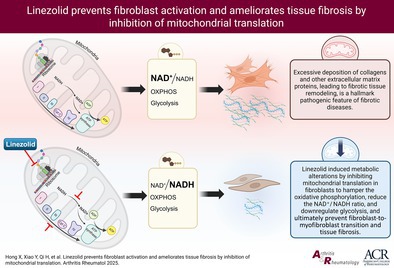

## Graphical abstract

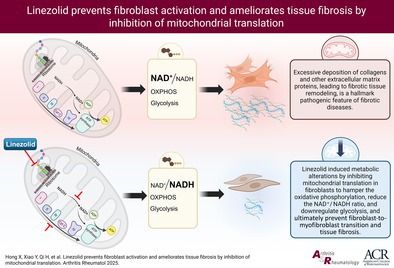

## INTRODUCTION

Excessive deposition of collagens and other extracellular matrix (ECM) proteins, leading to fibrotic tissue remodeling, is a hallmark pathogenic feature of fibrotic diseases such as systemic sclerosis (SSc) or sclerodermatous chronic graft‐versus‐host disease (sclGvHD).^1^ The aberrant ECM deposition interferes with organ physiology, leading to impairment of organ function, with subsequent morbidity and death for patients.^2^

Ribosome‐targeting antibiotics such as linezolid exert their antibiotic effects by interfering with the function of bacterial ribosomes and thus preventing bacterial protein synthesis. Because the structure of eukaryotic mitochondrial ribosomes (mitoribosomes) is similar to that of bacterial ribosomes, ribosome‐targeting antibiotics inhibit protein translation mediated by mitoribosomes, without affecting protein translation mediated by cytosolic ribosomes. The electron transport chain complex (ETC) biogenesis is dependent on the cooperation between the cytosolic and mitochondrial protein synthesis; thus, by specifically inhibiting mitochondrial translation, ribosome‐targeting antibiotics can induce an imbalance between the nuclear and mitochondrial synthesis of ETC subunits. This imbalance has been previously shown to prevent interleukin‐17 (IL‐17) production by Th17 cells or adipokine secretion by adipocytes.^3^, ^4^

Mitochondrial damage has been previously reported in several fibrotic diseases, including SSc.^5^, ^6^ Mitochondrial damage contributes to the presence of factors that induce mitochondrial stress, such as hypoxia and chronic inflammation, as well as the downregulation of factors that could counteract this effect, such as the transcription factor TFAM.^6^ Myofibroblasts rely predominantly on glycolysis as a source of ATP to exert their pathologic functions.^7^, ^8^ However, functional mitochondria are not only required for generation of ATP but also for the regeneration of the oxidized forms of electron carriers (NAD+ and FAD). Because key glycolytic enzymes (ie, GAPDH and lactate dehydrogenase), use NAD+ as a co‐factor,^9^ the reduced availability of NAD+ can limit glycolysis. Furthermore, inhibition of mitochondrial translation might further aggravate the metabolic stress of myofibroblasts and thereby prevent synthesis of glycolytic enzymes.

We reasoned that, in the context of pre‐existing mitochondrial damage, the ribosome‐targeting antibiotic linezolid might further aggravate the mitochondrial dysfunction to limit the glycolytic activity in myofibroblasts. Thus, by interfering with the high glycolytic requirements of myofibroblasts, ribosome‐targeting antibiotics might prevent fibroblast‐to‐myofibroblast differentiation and fibrotic remodeling in SSc.

## MATERIALS AND METHODS

### Induced pluripotent stem cells reprogramming

Reprogramming of fibroblasts to induced pluripotent stem cells (iPSCs) was performed as previously described.^10^, ^11^ Human dermal fibroblasts isolated from skin biopsies of patients with SSc in passages three or four were cultured in six‐well plates with Dulbecco's Modified Eagle Medium (DMEM)/F12 (31330‐038; Gibco, Carlsbad, CA) supplemented with 10% fetal bovine serum (FBS) (10270‐016; Thermo Fisher Scientific, Carlsbad, CA). iPSCs were generated from fibroblasts using the CytoTune iPS 2.0 Sendai Reprogramming Kit (A16517; Thermo Fisher Scientific), according to the manufacturer's instructions. Briefly, cell lines were transduced with nonintegrating Sendai virus containing four reprogramming factors: c‐MYC, KLF4, OCT3/4, and SOX2. When cell cultures reached 70% to 80% confluency, iPSCs were washed once with DMEM/F12 (31331‐028; Gibco) and incubated with Gentle Cell Dissociation Reagent ReLeSR (5872; STEMCELL Technologies, Vancouver, BC, Canada) for five minutes at room temperature for passaging. Gentle Cell Dissociation Reagent was aspirated, and StemMACS iPS‐Brew XF (130‐104‐368; Miltenyi Biotec) supplemented with 100 U/mL penicillin/streptomycin (15140‐122; Life Technologies, Carlsbad, CA) was added. Corning Cell Lifter was used to detach human iPSCs from the cell culture plate. iPSCs were transferred to a new Geltrex‐coated plate. Cells with a TRA1‐60–positive rate exceeding 75% were selected for further passage and culture. Two clones were used from each donor.

### iPSC culture

IPSCs were cultured in mTeSR Plus Basal Medium (100‐0274; STEMCELL Technologies) with daily medium changes. Once cells reached 70% to 80% confluence, they were used directly for experiments. For differentiation, the purity of cells was assessed, and differentiated cells were removed before proceeding.

### Macrophage differentiation

Macrophage differentiation from SSc iPSC was performed as described.^12^ iPSCs were detached using Accutase (A1110501; Gibco) and seeded into AggreWell800 plate (34821; STEMCELL Technologies) in mTeSRPlus medium with 10 μM ROCK inhibitor (Y0503‐1MG; Sigma‐Aldrich, Steinheim, Germany). On day 2, the medium was replaced with mTeSR medium containing 50 ng/mL BMP4 (Y0503‐1MG; Sigma‐Aldrich), 50 ng/mL VEGF (100‐20; PeproTech, Hamburg, Germany), and 20 ng/mL SCF (255‐SC; Bio‐techne, Wiesbaden, Germany) for four to five days. Newly formed embryoid bodies were transferred to 1% Geltrex–coated (A1413302; Thermo Fisher Scientific) six‐well plates in X‐VIVO 15 (BE02‐053Q; Lonza, Basel, Switzerland) with 100 ng/mL M‐CSF (574806; BioLegend, San Diego, CA), 25 ng/mL IL‐3 (200‐03; PeproTech), 2 mM Glutamax (255030‐024; Life Technologies), 50 μg/mL mercaptoethanol (21985‐023; Gibco), and 1% antibiotics (15140‐122; Life Technologies). Medium was refreshed at week 1, followed by biweekly 50% changes. Hematopoietic cells appeared within two to three weeks. Nonadherent cells were centrifuged (300 g, three minutes) and resuspended in X‐VIVO 15 (BE02‐053Q; Lonza) with 100 ng/mL M‐CSF (574806; BioLegend), 2 mM Glutamax (255030‐024; Life Technologies), and 1% antibiotics (15140‐122; Life Technologies). Macrophages matured over seven days and were subsequently collected for further use.

### l‐homopropargylglycine assay

l‐homopropargylglycine (HPG) assays were performed following an established protocol.^13^ Fibroblasts were seeded in 96‐well plates at a density of 1,200 cells per well. After 24 hours of serum deprivation, cells were stimulated with 10 ng/mL TGFβ (100‐21C; PeproTech), 1 ng/mL IL‐1β (200‐01B; PeproTech), or 10 ng/mL IL‐4 (200‐04; PeproTech) and treated with 100 μM linezolid (HY‐10394; MedChemExpress, MCE, Monmouth Junction, New Jersey) or vehicle for seven days. The culture medium was changed to methionine‐free (met‐free) DMEM, which included DMEM (21013‐024; Gibco), 48 μg/mL l‐cystine dihydrochloride (J62292.14; Thermo Fisher Scientific), 4.08 mM l‐alanyl‐l‐glutamine solution (STA‐B; Capricorn Scientific, Eschwege, Germany), and 10% FBS (10500‐064; Gibco), met‐free DMEM + 100 μg/mL anisomycin (ANS) (HY‐18982; MCE), or met‐free DMEM + 100 μg/mL ANS + 100 μM HPG (CLK‐1067‐25; Jena Bioscience, Jena, Germany) for three hours. Then, cells were rinsed with 100 μL of prewarmed phosphate‐buffered saline (PBS) and fixed with 4% paraformaldehyde in 100 μL for 15 minutes. Following fixation, the cells were permeabilized using 0.1% (volume/volume) Triton X‐100 in 100 μL of PBS for five minutes. To label nascent proteins, the cells were incubated for 30 minutes in 100 μL of click reaction buffer containing 1× Click‐iT cell reaction buffer, 2 mM copper sulfate, 1× Click‐iT cell buffer additive, and 1 μM azide‐conjugated cyanine 3 (CLK‐046‐1; Jena Bioscience), following the protocol provided with the Click‐iT Cell Reaction Buffer Kit (C10269; Thermo Fisher Scientific). After this step, the cells were washed with 100 μL of blocking buffer. For mitochondrial immunostaining, the cells were incubated with 100 μL of blocking buffer containing 1 μL of Alexa Fluor 647–conjugated anti‐TOM20 antibody (ab209606; Abcam) and 0.1 μL of DAPI for 1 hour at 4°C. Finally, the cells were washed twice with 200 μL of PBS and mounted using 100 μL of 50% glycerol in PBS. HPG images were captured using the CellInsight CX5 (Thermo Fisher Scientific) and automatically analyzed for average fluorescence intensity with HCS Studio Cell Analysis Software.

### Murine sclGvHD

The B10.D2→BALB/c (H‐2d) minor histocompatibility antigen‐mismatched model was used as previously described.^14^ Female BALB/c (H‐2d) mice were purchased from Janvier Labs (Le Genest Saint Isle, France), and male B10.D2 (H‐2d) mice were purchased from The Jackson Laboratory (Bar Harbor, ME). Mice were housed under pathogen‐free conditions at the University of Erlangen‐Nuremberg. Bone marrow cells were isolated from tibial and femoral bones by flushing with PBS, whereas splenocytes were obtained by gently mashing the spleen with a syringe piston, followed by filtration through 70‐μm meshes (BD Biosciences) and red blood cell lysis buffer (420301; BioLegend, Koblenz, Germany). Cells were kept on ice before transplantation. BALB/c recipients received total body irradiation (7 Gy) and, six hours later, were injected with 2 × 10^6^ bone marrow cells and 4.5 × 10^6^ splenocytes from syngeneic BALB/c or allogeneic B10.D2 donors via the tail vein. Starting 21 days post‐transplantation, after chronic graft‐versus‐host disease (cGvHD) symptoms appeared in allogeneically transplanted mice, 4 mg/day linezolid or vancomycin (HY‐B0671; MCE) was administered. Outcomes were assessed seven weeks after transplantation. All procedures adhered to international animal welfare guidelines and were approved by the government of Unterfranken, Würzburg, Germany and the University of Erlangen‐Nuremberg's animal care committee.

### Three‐dimensional SSc skin equivalents

Three‐dimensional SSc skin equivalents (SScSE) were adapted from previously described protocols^15^, ^16^ to incorporate SSc iPSC‐derived macrophages. Primary dermal fibroblasts isolated from patients with SSc (0.2 × 10^6^ cells) were combined with an equal number of iPSCs‐derived macrophages, which were reprogrammed from the primary dermal fibroblasts of patients with SSc (see above). The cells were suspended in a neutralization buffer consisting of 232.5 mL DMEM/F‐12 (SLM‐202‐B; Merck Millipore, Darmstadt, Germany), 7.5 mL FBS (10270‐106; Thermo Fisher Scientific), 7.5 mL 3M HEPES (H3375‐25G; Sigma‐Aldrich), and 2.5 mL 5 mg/mL chondroitin sulfate (C4384‐250MG; Sigma‐Aldrich). A total of 500 μl of this mixture was prepared using one‐third of the cells in neutralization buffer and two‐thirds rat tail collagen (354249; Corning). The mixture was seeded onto inserts equipped with 8‐μm porous membranes (665638; Greiner Bio‐One, Frickenhausen, Germany). The populated inserts were incubated at 37°C for 45 to 60 minutes to allow the dermal layer to form, after which fibroblast medium was added and left overnight. On the following day, 0.5 × 10^6^ keratinocytes were seeded onto the constructs to create the epidermal layer. The skin equivalents were then cultured using EpiLife medium (MEPI500CA; Thermo Fisher Scientific) supplemented with 1% human keratinocyte growth supplement (S‐001‐5; Thermo Fisher Scientific), 1% penicillin/streptomycin (15140‐122; Life Technologies), and 1.44 mM calcium chloride (CaCl_2_) (CN93.1; Carl ROTH, Karlsruhe, Germany). After 24 hours, the models were gently detached using 27‐gauge needles. The medium on the upper layer was carefully removed to allow direct air exposure for the keratinocytes, whereas the medium was replaced with EpiLife medium supplemented with 1% human keratinocyte growth supplement, 1% penicillin/streptomycin, 1.44 mM CaCl_2_, 10 ng/mL Keratinocyte Growth Factor (K1757‐10UG; Sigma‐Aldrich), and 0.125 mM l‐ascorbic acid 2‐phosphate (49752‐10G; Sigma‐Aldrich). The skin equivalents were treated on the second day with either 10 ng/mL TGFβ or TGFβ + 100 μM linezolid.

### Precision‐cut skin slices

Precision‐cut skin slices (PCSS) from patients with SSc were generated as previously described.^15^ Skin biopsies were obtained from patients with SSc after obtaining informed consent. The biopsies were embedded in 3% low‐melting agarose (16520100; Thermo Fisher Scientific) dissolved in 0.9% sodium chloride. Alabama R&D Tissue Slicer (Alabama Research and Development) was used to generate the 350‐μm thickness of PCSS. The sections were then transferred into 24‐well plates containing prewarmed Minimum Essential Medium (51200‐087; Life Technologies), supplemented with 11.34 mM glucose (182811; Komtur Pharmaceuticals, Freiburg, Germany), 26 mM NaHCO_3_ (S8761; Sigma‐Aldrich), 25 mM HEPES (15630; Gibco), 1 mM sodium pyruvate (11360; Gibco), 2 mM GlutaMAX (35050; Gibco), 1% penicillin/streptomycin, and amphotericin. Cultures were maintained at 37°C in 5% carbon dioxide and atmospheric oxygen. Starting from the second day, PCSS were treated with 100 μM linezolid or with DMSO, the vehicle of linezolid. After 48 hours, the PCSS samples were collected for RNA‐sequencing (RNAseq) analysis and immunofluorescence staining. Comprehensive descriptions of the materials and methods are available in the online supplementary materials due to word count limitations in the main text.

### Data statement

The RNAseq data are publicly available on the GEO database (GEO: https://www.ncbi.nlm.nih.gov/geo/, GSE310202). Further supporting data are available from the corresponding author upon reasonable request.

## RESULTS

### Inhibition of fibroblast‐to‐myofibroblast transition and collagen deposition in cultured human dermal fibroblasts by linezolid

We aimed to evaluate whether treatment with linezolid can prevent fibroblast‐to‐myofibroblast transition induced by TGFβ in cultured human fibroblasts. To exclude potential cytotoxic effects of linezolid at the concentration used, we first performed a CCK‐8 assay in fibroblasts treated with increasing doses of linezolid (0–200 μM). No significant reduction in cell viability was observed up to 100 μM (Supplementary Figure 1), the concentration used in all subsequent experiments.

**Figure 1:**
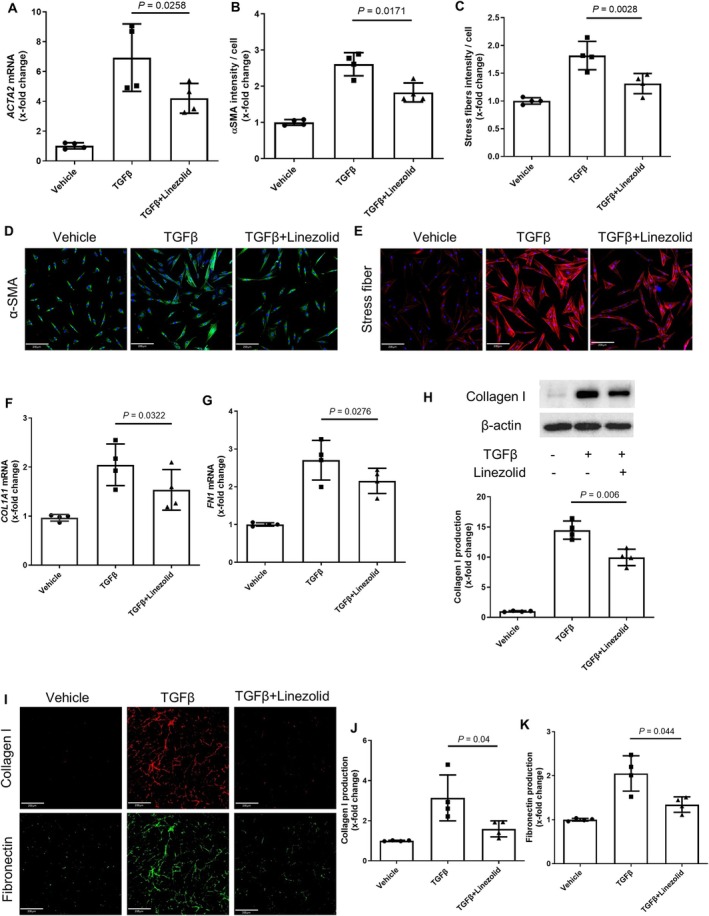
Linezolid ameliorates TGFβ‐induced fibroblast activation and collagen deposition in cultured human dermal fibroblasts. (A) Expression levels of *ACTA2* mRNA. (B–E) Quantification of stress fiber formation (B) and αSMA expression (C) and representative immunofluorescence images showing αSMA (D) and stress fibers (E) in human dermal fibroblasts (scale bar = 200 μm). (F–G) Expression levels of *COL1A1* and *FN1* mRNA. (H) Protein expression levels of collagen type 1. Representative Western Blot and quantification were included. (I–K) Representative immunofluorescence images (I) and quantification of collagen type 1 (J) and fibronectin (K) deposited by human fibroblasts, Data are presented as mean ± SD from four independent biologic replicates per group. Statistical analysis was performed using a paired *t*‐test. mRNA, messenger RNA.

Treatment with linezolid ameliorated TGFβ‐induced *ACTA2* messenger RNA (mRNA) upregulation, αSMA protein expression, stress fibers formation, and gel contraction, all hallmarks of myofibroblast differentiation (Figure 1A–1E; Supplementary Figure 2). Furthermore, treatment with linezolid reduced TGFβ‐induced *COL1A1* and *FN1* mRNA and of collagen type 1 and fibronectin protein upregulation (Figure 1F–1K).

RNAseq analysis revealed that incubation with linezolid induced profound transcriptomic changes in TGFβ‐stimulated fibroblasts (Supplementary Figure 3A–3B). Pathway enrichment analysis using the Gene Ontology (GO) database and gene set enrichment analysis (GSEA) showed de‐enrichment of pathways related to ECM remodeling, such as “extracellular matrix organization,” and of profibrotic signaling pathways, such as “canonical WNT signaling pathway” (Supplementary Figure 3C–3D).

### Amelioration of fibrotic remodeling in an SScSE model by linezolid

**Figure 2:**
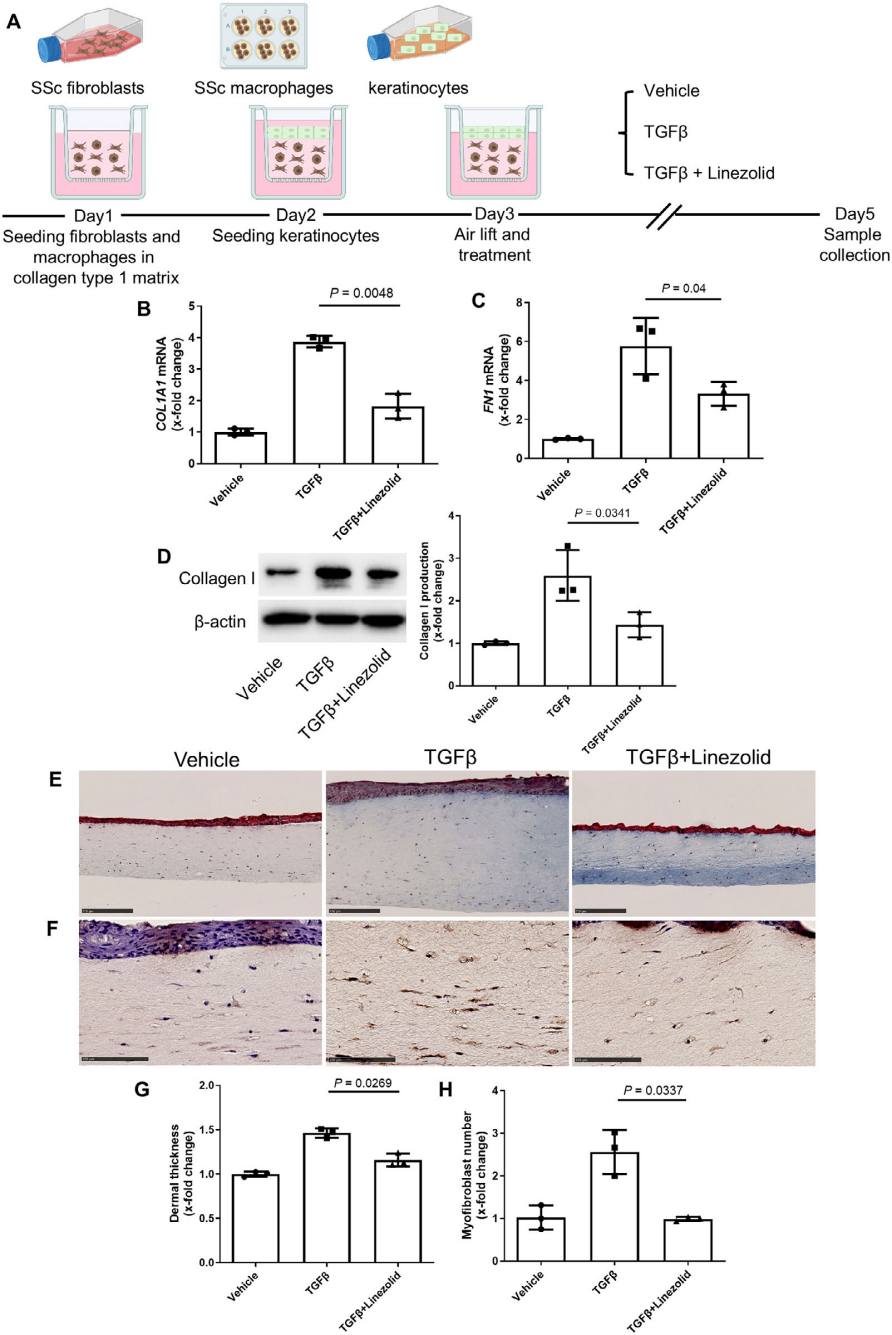
Linezolid inhibits TGFβ‐induced fibrotic transformation of three‐dimensional SSc skin equivalents. (A) Schematic representation of the experimental design involving SScSE, created with BioRender.com. (B–C) Expression levels of *COL1A1* and *FN1* mRNA. (D) Protein expression levels of collagen type 1. Representative Western blot and quantification were included. (E) Representative Masson's trichrome staining of SScSE (scale bar = 250 μm). (F) Representative immunohistochemical staining of αSMA (scale bar = 100 μm). (G–H) Quantification of dermal thickness and myofibroblast count in SScSE. Data are presented as mean ± SD from three independent biologic samples per group. *P* values determined by paired *t*‐test. mRNA, messenger RNA; SSc, systemic sclerosis; SScSE, SSc skin equivalents. Color figure can be viewed in the online issue, which is available at http://onlinelibrary.wiley.com/doi/10.1002/art.43440/abstract.

We next aimed to evaluate whether treatment with linezolid can prevent fibroblast‐to‐myofibroblast transition and collagen deposition in three‐dimensional SScSE. This model is composed of an epidermal layer and a dermal component with SSc fibroblasts and SSc macrophages and exposed to TGFβ to recapitulate key pathogenic cellular players, cell–cell interactions, and profibrotic stimuli in SSc (Figure 2A). Incubation with linezolid prevented TGFβ‐induced upregulation of *COL1A1* and *FN1* mRNAs and of collagen type 1 protein in the SScSE (Figure 2B–2D). Furthermore, linezolid prevented TGFβ‐induced increases in dermal thickness and myofibroblast counts in SScSE (Figure 2E–2H).

Given the strong effects of linezolid in the SScSE with an almost complete abrogation of TGFβ‐induced fibroblast‐to‐myofibroblast transition and collagen deposition, we reasoned that linezolid might target not only fibroblasts but also macrophages. RNAseq analysis demonstrated that incubation with linezolid induced transcriptomic changes in TGFβ‐exposed macrophages, with 337 differentially expressed genes (DEGs) (Supplementary Figure 4A–4B). GO pathway enrichment analysis and GSEA revealed that linezolid modulated biologic processes related to macrophage activation, fibroblast‐to‐myofibroblast transition, and collagen deposition (eg, “collagen formation” or “actin filament organization”) (Supplementary Figure 4C–4D). Furthermore, linezolid induced de‐enrichment of several terms related to metabolic pathways or mitochondrial function, such as “glycolysis” or “mitochondrial ATP synthesis coupled electron transport” (Supplementary Figure 4C–4D).

To further confirm the effects of linezolid on macrophages, we assessed the mRNA expression of the macrophage genes *TNFα*, *IL‐6*, *MMP1*, and *MMP9* in the SScSE exposed to TGFβ. Linezolid markedly reduced the expression levels of all four genes (Supplementary Figure 5A–5D), providing evidence that linezolid modulates macrophage activity in situ in the context of skin fibrosis in SSc, which might contribute to its antifibrotic effects.

### Amelioration of experimental cGvHD by linezolid

**Figure 3:**
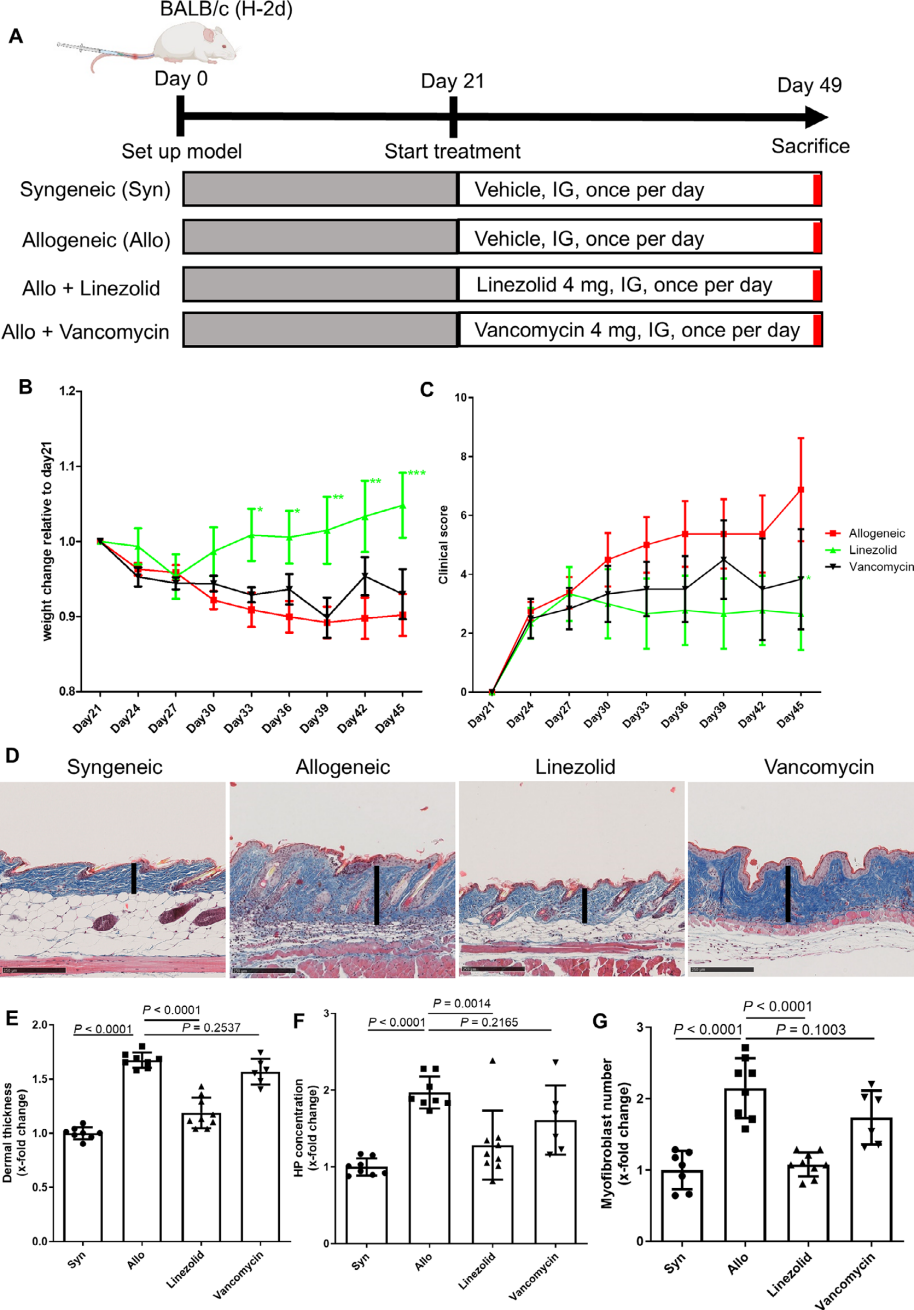
Treatment with linezolid ameliorates clinical manifestations and skin fibrosis in murine sclGvHD. (A) Schematic representation of the experimental design involving the murine sclGvHD model, created using BioRender.com. (B–C) Effects of linezolid or vancomycin on weight loss (B) and clinical composite score (C) in allogeneically transplanted mice. (D) Representative Masson's trichrome staining of skin from sclGvHD mice (scale bars = 250 μm). (E–G) Quantification of dermal thickness (E), hydroxyproline content (F), and myofibroblast counts (G) in the dermis of sclGvHD mice. Data are shown as mean ± SD, with n = 6 to 9 biologic samples per group. Statistical significance was determined using two‐way ANOVA with Dunnett's test for weight loss and clinical scores and one‐way ANOVA with Tukey's test for dermal thickening, hydroxyproline content, and myofibroblast counts. *0.05 > *P* > 0.01, **0.01 > *P* > 0.001, ***P < 0.001. ANOVA, analysis of variance; HP, hydroxyproline; sclGvHD, sclerodermatous chronic graft‐versus‐host disease. Color figure can be viewed in the online issue, which is available at http://onlinelibrary.wiley.com/doi/10.1002/art.43440/abstract.

We next aimed to evaluate the antifibrotic effects of linezolid in vivo. We performed the B10.D2 (H‐2d) → BALB/c (H‐2d) model of sclGvHD, a model with multiorgan inflammation‐dependent fibrotic tissue remodeling, including involvement of the skin and lungs, resembling an aggressive form of diffuse cutaneous SSc.^17^ We treated mice that underwent allogeneic stem cell transplantation (alloSCT) with linezolid starting at day 21 after SCT, after first clinical signs of cGvHD became clinically manifest (Figure 3A). We included an experimental group of alloSCT mice treated with vancomycin, an antibiotic with a similar spectrum to linezolid^18^ but without inhibitory effects on mitochondrial translation (Figure 3A). This allowed us to account for a potential contribution of the antibiotic effects of linezolid to the overall effects on sclGvHD.

Treatment with linezolid ameliorated weight loss and clinical signs of cGvHD in alloSCT mice, whereas treatment with vancomycin demonstrated only mild trends toward protective effects (Figure 3B–3C). Furthermore, treatment with linezolid strongly reduced dermal thickness, hydroxyproline concentration, and number of myofibroblasts in the skin of alloSCT mice to levels comparable to syngeneically transplanted mice, whereas treatment with vancomycin had only mild effects (Figure 3D–3G). Treatment with linezolid did not only ameliorate skin fibrosis but also demonstrated antifibrotic effects in the lungs of GvHD mice, with reduced Ashcroft scores, lung hydroxyproline content, and myofibroblast counts (Supplementary Figure 6A–6D).

Both linezolid and vancomycin demonstrated strong anti‐inflammatory effects in the colon of alloSCT mice without affecting the numbers of LRG5‐positive intestinal stem cells (Supplementary Figure 6E–6G). This may indicate that the intestinal effects of linezolid are at least in part driven by its antibiotic effects on the intestinal microbiome, in contrast to its antifibrotic effects in skin and lung fibrosis, in which antibiotic effects can only partially explain the phenotype.

### Reversal of profibrotic and proinflammatory pathways in experimental sclGvHD by linezolid

**Figure 4:**
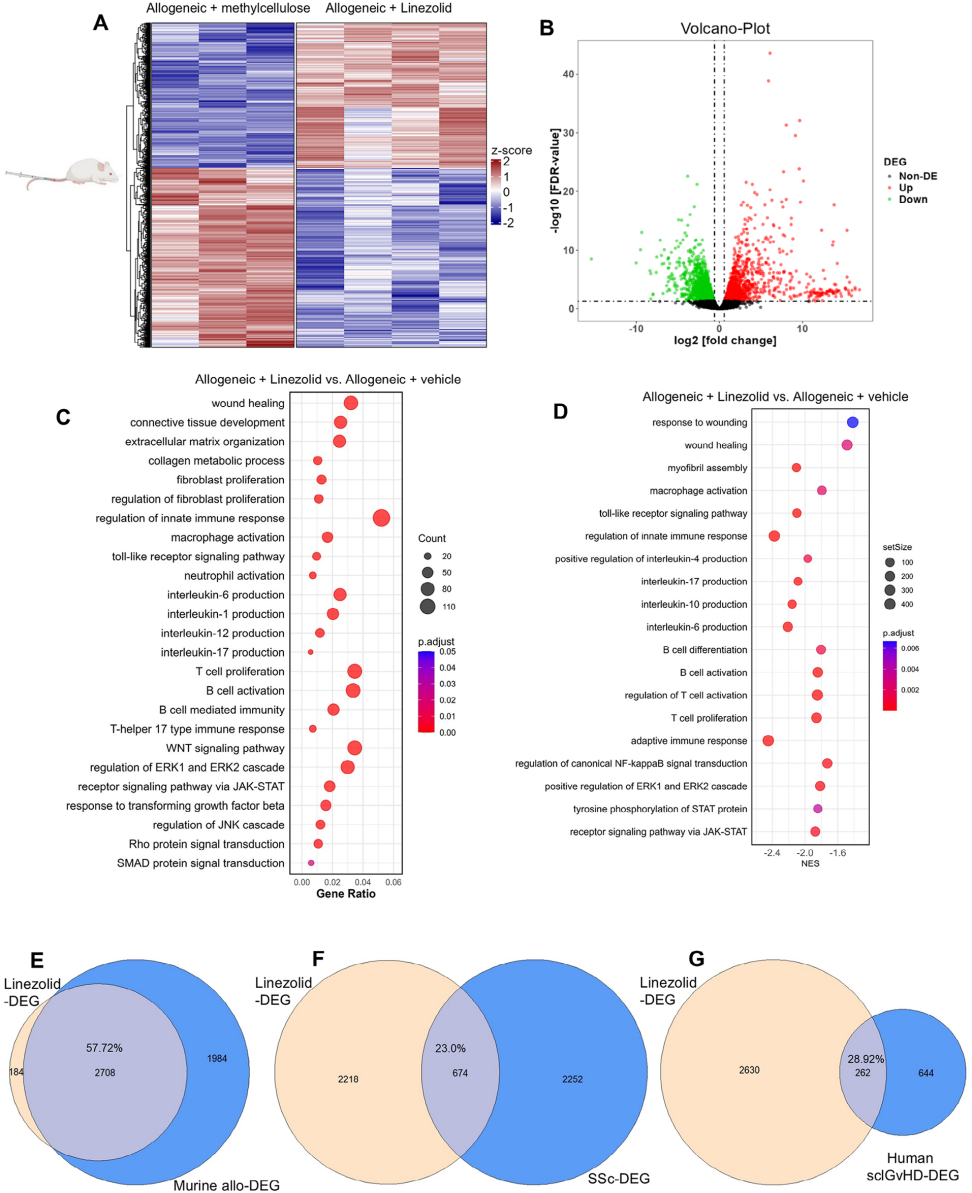
Linezolid down‐regulates profibrotic and proinflammatory gene expression programs in the skin of sclGvHD mice. (A–B) Heatmap (A) and volcano plot (B) displaying DEGs in allogeneically transplanted mice treated with linezolid (n = 4) compared with the vehicle‐treated group (n = 3). (C–D) Bubble plots highlighting significantly enriched biologic processes related to inflammation and fibrosis in the skin of allogeneically transplanted sclGvHD mice treated with linezolid vs vehicle, based on GO analysis (C) and GSEA (D). (E–G) Intersection of Line‐DEGs (DEGs in the skin of allo‐GvHD treated with linezolid vs vehicle) with murine allo‐DEGs (DEGs in the skin allogeneically vs syngeneically transplanted mice) (E), with SSc‐DEGs (DEGs in the skin of patients with SSc vs controls) (F), and with human sclGvHD‐DEGs (DEGs in the skin of patients with sclGvHD vs controls). The percentages were calculated based on the number of overlapping DEGs relative to the total number of murine allo‐DEGs (E), of SSc‐DEGs (F), or of sclGvHD‐DEGs (G). allo, allogenic; DEG, differentially expressed genes; FDR, false discovery rate; GO, gene ontology; GSEA, gene set enrichment analysis; SSc, systemic sclerosis; sclGvHD, sclerodermatous graft‐versus‐host disease. Color figure can be viewed in the online issue, which is available at http://onlinelibrary.wiley.com/doi/10.1002/art.43440/abstract.

RNAseq analysis demonstrated that treatment with linezolid had profound transcriptomic effects in the skin of alloSCT mice, with 1,564 down‐regulated and 1,328 up‐regulated DEGs (Figure 4A–4B). GO pathway enrichment analysis and GSEA demonstrated de‐enrichment of biologic processes related to fibrosis (eg, “collagen metabolic process”) and to innate and adaptive immune responses (eg, “regulation of innate immune response,” “macrophage activation,” “B cell activation,” “T cell proliferation,” or “T‐helper 17 type immune response”) (Figure 4C–4D). Immunofluorescence staining demonstrated that mice that underwent alloSCT had markedly increased skin densities of CD45+ leukocytes, as well as of leukocyte subsets, CD3+ T cells, B220+ B cells, and CD68+ macrophages, whereas these effects were prevented by treatment with linezolid (Supplementary Figure 7), confirming the immunomodulatory effects of linezolid inferred by RNAseq.

Furthermore, RNAseq analysis showed that key pathogenic signaling pathways in SSc and GvHD, such as TGFβ, SMAD, JNK, ERK, or JAK‐STAT, and ILs, such as IL‐1, IL‐4, IL‐6 or IL‐17, were de‐enriched upon linezolid treatment (Figure 4C–4D). Treatment with linezolid also modulated metabolic pathways, with de‐enrichment of GSEA pathways related to glycolysis and reactive oxygen species production (Supplementary Figure 8).

Treatment with linezolid reverted 58% of the murine DEGs in allogeneic vs syngeneic mice (allo‐DEGs) (Figure 4E). We further data mined publicly available RNAseq datasets from the skin of patients with SSc or sclGvHD to evaluate the overlap between the DEGs in the skin of sclGvHD mice treated with linezolid vs vehicle and DEGs in the skin of patients with SSc vs controls (SSc‐DEGs) or DEGs in the skin of patients with sclGvHD vs controls (sclGvHD‐DEGs). Despite species‐specific differences, treatment with linezolid reverted 23% of the human SSc‐DEGs and 29% of the human sclGvHD‐DEG (Figure 4F).

Although the transcriptome of alloSCT mice treated with linezolid closely resembled that of syngeneic mice, treatment with vancomycin induced only mild transcriptomic changes and had a transcriptome closer to allogeneic mice and more distant from syngeneic mice (Supplementary Figure 9A–9B). Functional enrichment analysis showed that treatment with linezolid can revert several pathogenically relevant biologic processes and pathways that are not modulated by vancomycin (Supplementary Figure 9C), highlighting that the antibiotic effects could only partially explain the antifibrotic and anti‐inflammatory effects of linezolid. Consistently, treatment with vancomycin reverted much smaller fractions of the murine allo‐DEGs, the SSc‐DEGs, or the human sclGvHD‐DEG than linezolid (Supplementary Figure 9D–9F).

### Reversal of profibrotic and proinflammatory pathways in SSc skin by linezolid

**Figure 5:**
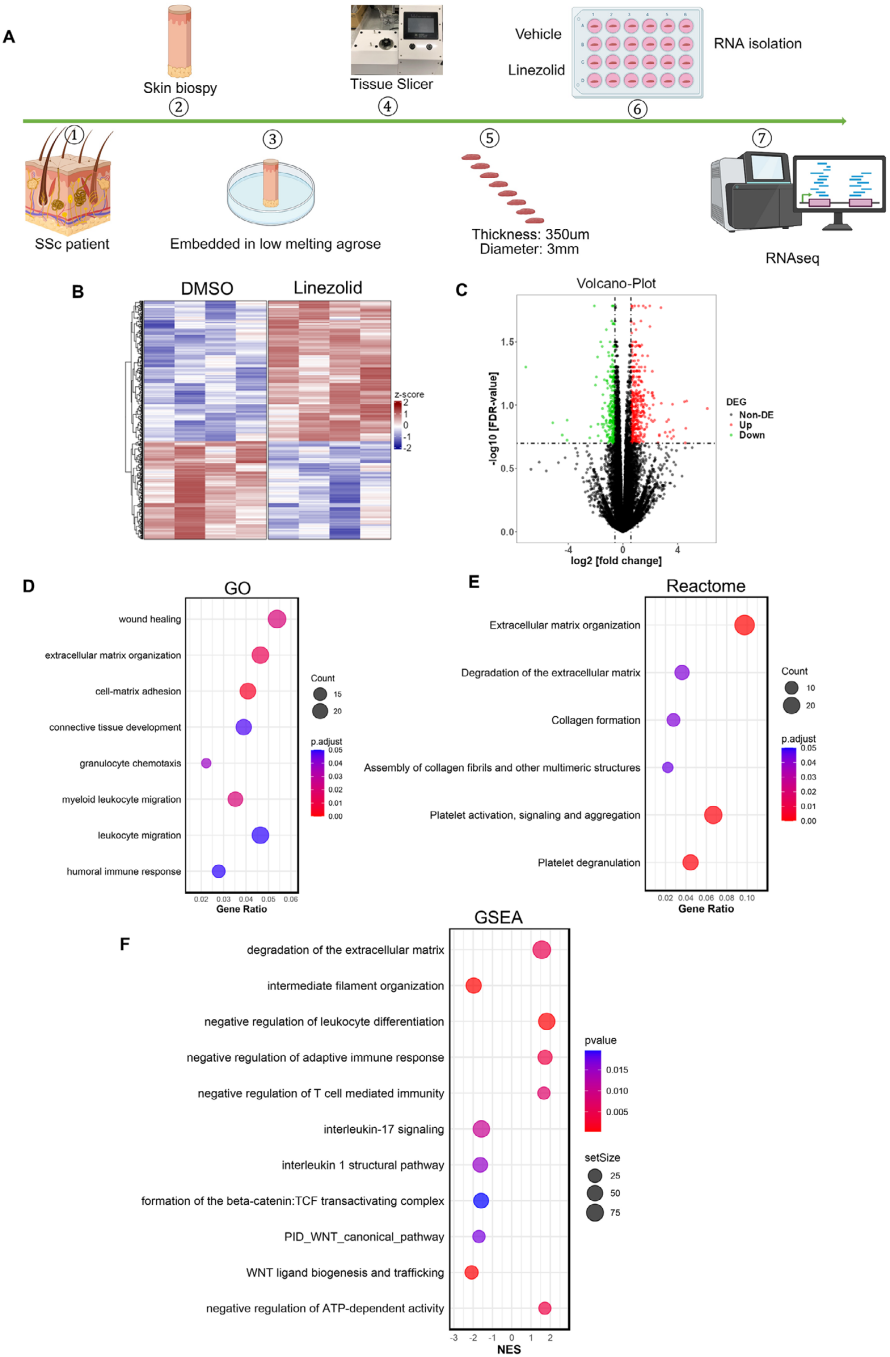
Treatment with linezolid reverts an aberrant SSc gene expression profile in the skin of patients with SSc. (A) Schematic representation of the experimental setup for the PCSS model, created using BioRender.com. (B–C) Heatmap (B) and volcano plot (C) displaying DEGs between SSc‐PCSS samples treated with linezolid and the vehicle control. (D–F) Bubble plots highlighting significantly enriched biologic processes associated with inflammation and fibrosis in PCSS samples treated with linezolid vs vehicle, based on GO analysis (D), Reactome pathway analysis (E), and GSEA (F). DEG, differentially expressed gene; FDR, false discovery rate; GO, gene ontology; GSEA, gene set enrichment analysis; PCSS, precision‐cut skin slices; RNAseq, RNA sequencing; SSc, systemic sclerosis. Color figure can be viewed in the online issue, which is available at http://onlinelibrary.wiley.com/doi/10.1002/art.43440/abstract.

We further aimed to evaluate the effects of linezolid directly in the skin of patients with SSc. We generated SSc‐PCSS, which includes all cell types found in human skin in situ, and treated them with linezolid or vehicle in vitro (Figure 5A). Incubation with linezolid induced transcriptomic changes in the SSc skin, with 651 DEGs compared with SSc‐PCCS with vehicle treatment (Figure 5B–5C). Pathway enrichment analysis by GO or Reactome, as well as GSEA, demonstrated that incubation with linezolid regulated the expression of several pathogenically relevant pathways in SSc, including “extracellular matrix organization,” “collagen formation,” “interleukin‐1,” “interleukin‐17 signaling,” or “WNT canonical pathway” (Figure 5D–5F). Furthermore, incubation with linezolid reduced the number of αSMA^+^/P4Hβ^+^ myofibroblasts in SSc‐PCSS compared with vehicle treatment (Supplementary Figure 10), confirming its inhibitory effects on fibroblast‐to‐myofibroblast transition in situ.

### Inhibition of mitochondrial translation in human fibroblasts by linezolid

**Figure 6:**
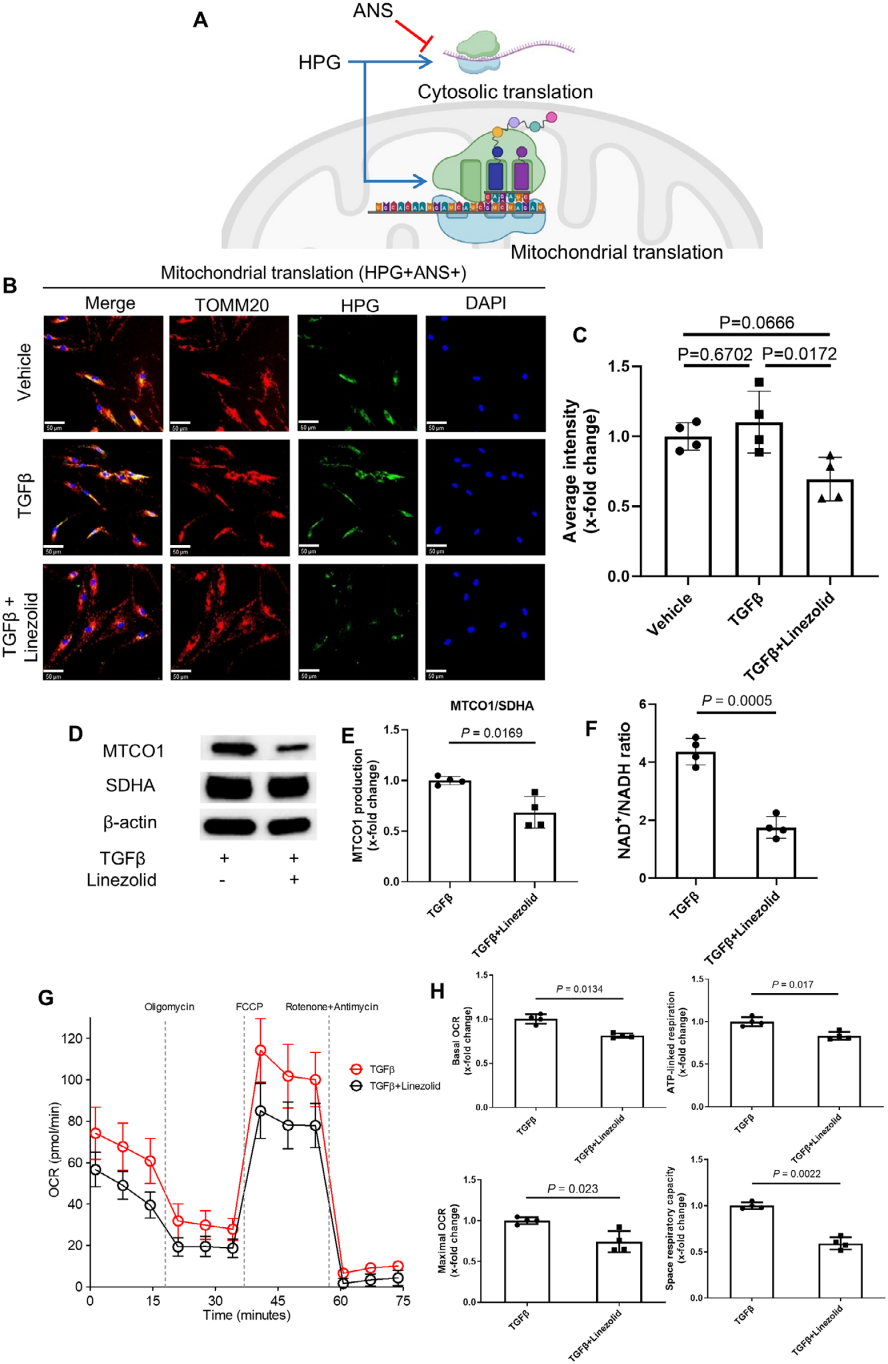
Linezolid inhibits mitochondrial translation, decreases the NAD^+^/NADH ratio, and impairs mitochondrial respiration in human fibroblasts. (A) Schematic representation of the incorporation of l‐homopropargylglycine (HPG), a methionine analog, in nascent cytosolic and mitochondrial proteins and the effects of anisomycin (ANS) in blocking cytosolic translation and allowing incorporation of HPG exclusively in mitochondrial proteins. The figure was created using BioRender.com. (B–C) Representative immunofluorescence images of HPG (green) and the mitochondrial protein TOMM20 (red) (B) and quantification of average intensity of HPG (C), showing incorporation of HPG in mitochondrial nascent proteins in cultured human fibroblasts (scale bars = 50 μm). (D–E) Representative Western blot (D) and quantification of MTCO1 protein levels (E). (F) NAD^+^/NADH ratio in cultured human fibroblast. (G–H) Seahorse assay results, including oxygen consumption rate (OCR) curves (G) and quantification of basal OCR, ATP‐linked respiration, maximal OCR, and spare respiratory capacity (H). Data are presented as mean ± SD, with n = 4 independent biologic samples per group. *P* values determined by one‐way ANOVA with Tukey's test or paired *t*‐test. ANOVA, analysis of variance; FCCP, carbonyl cyanide‐p‐trifluoromethoxyphenylhydrazone. Color figure can be viewed in the online issue, which is available at http://onlinelibrary.wiley.com/doi/10.1002/art.43440/abstract.

To evaluate whether inhibition of mitochondrial translation underlies the antifibrotic effects of linezolid, we employed a protein synthesis assay that traces the incorporation of HPG, a methionine analog, in nascent proteins. We treated fibroblasts with ANS, which inhibits specifically cytosolic translation and allows evaluation of HPG uptake in nascent mitochondrial proteins and, thus, evaluation of mitochondrial translation decoupled from the cytosolic translation (Figure 6A; Supplementary Figure 11).^19^ Treatment with ANS decreased the global HPG uptake in human fibroblasts, with incorporation of HPG specifically in mitochondria (Supplementary Figure 11). Incubation with linezolid markedly decreased the HPG uptake in healthy and SSc fibroblasts exposed to either TGFβ, IL‐1β, or IL‐4 and treated with ANS and, thus, mitochondrial translation (Figure 6B–6C; Supplementary Figure 12). In line with these results, treatment with linezolid markedly reduced the levels of the mitochondrial protein MTCO1 expression (Figure 6D–6E). Furthermore, treatment with linezolid further reduced the NAD^+^/NADH ratio (Figure 6F) and impaired the respiratory flux in fibroblasts treated with TGFβ, with reduced basal oxygen consumption rate (OCR), maximal OCR, ATP‐linked respiration, and spare respiratory capacity (Figure 6G–6H), highlighting that interference with mitochondrial translation in TGFβ‐stimulated fibroblasts leads to net functional mitochondrial impairment.

## DISCUSSION

Although several reports highlight the critical need of metabolic reprogramming for fibroblast‐to‐myofibroblast transition, with mitochondrial dysfunction and up‐regulated glycolysis,^6^, ^7^, ^8^ no direct link has been described between dysregulation of mitochondrial translation and fibrotic tissue remodeling. Furthermore, no therapeutic approach targeting these metabolic abnormalities is currently available. We report here for the first time that inhibition of mitochondrial translation with linezolid can prevent fibroblast‐to‐myofibroblast transition and aberrant collagen deposition, thus offering therapeutic potential in fibrotic diseases such as SSc and sclGvHD.

We demonstrate that treatment with linezolid, a reserve ribosome‐targeting antibiotic approved for the treatment of multiresistant infections with Gram‐positive bacteria,^20^ can prevent the profibrotic effects of TGFβ in cultured dermal fibroblasts. Consistently, linezolid prevents myofibroblast transition and collagen deposition in human SScSE as a model that recapitulates the interactions of fibroblasts with macrophages, keratinocytes, and the surrounding ECM. We further report antifibrotic effects of treatment with linezolid in sclGvHD as a model recapitulating an aggressive form of diffuse SSc with lung fibrosis and intestinal involvement.^17^ Furthermore, treatment with linezolid reduced the expression of genes and pathways with direct relevance in the pathogenesis of SSc and sclGvHD in both a murine sclGvHD model and in PCSS of SSc skin. Thus, inhibition of mitochondrial translation by linezolid showed antifibrotic effects in several complementary murine and human model systems that resemble different aspects of SSc and sclGvHD. Of note, treatment with vancomycin, an antibiotic with a similar spectrum to linezolid, but without inhibitory effects on mitochondrial translation, demonstrated only mild effects in vivo, highlighting that the effects of linezolid cannot be explained by modulation of the intestinal microbiome.

In addition to its effects on fibroblast‐to‐myofibroblast differentiation, inhibition of macrophage activation may also contribute to the antifibrotic effects of linezolid. Our RNAseq data from cultured macrophages exposed to TGFβ suggest that treatment with linezolid can revert TGFβ‐induced macrophage activation and macrophage‐driven fibroblast‐to‐myofibroblast transition. Biologic pathways related to macrophage activation were also de‐enriched in allogeneically transplanted mice upon linezolid treatment. Furthermore, inhibition of Th17 cytokine production might contribute to the antifibrotic effects of linezolid. Indeed, we found de‐enriched terms related to Th17 and IL‐17 production in the skin of cGvHD mice and in PCSS from patients with SSc. In line with this, a previous report demonstrated that linezolid can inhibit production of IL‐17 by Th17, with amelioration of a Th17‐driven autoimmune disease, by inhibition of mitochondrial translation.^3^

We further demonstrate that the linezolid‐induced interference with mitochondrial translation impairs the mitochondrial function with reduced OCR and respiratory capacity in human fibroblasts exposed to TGFβ. We hypothesize that these effects can prevent TGFβ‐induced collagen production primarily by interfering with the glycolytic pathway rather than by directly limiting ATP availability for collagen synthesis. Several reports demonstrated that myofibroblasts up‐regulate glycolysis and pyruvate fermentation to lactate even in the presence of sufficient oxygen (aerobic glycolysis) and thus require primarily glycolysis instead of oxidative phosphorylation (OXPHOS) for generation of ATP, as well as for generation of metabolites required for collagen synthesis.^7^, ^8^, ^21^ Indeed, inhibition of glycolytic enzymes demonstrated antifibrotic effects.^22^, ^23^, ^24^, ^25^, ^26^ However, the tricarboxylic acid (TCA) cycle and the ETC might still be indispensable in myofibroblasts for maintenance of a functional glycolysis (eg, by preventing the accumulation of NADH), and thus, inhibition of mitochondrial translation might secondarily impair glycolysis. In line with this, our RNAseq analyses in the skin of cGvHD mice demonstrate that linezolid inhibits the upregulation of glycolysis in allogeneically transplanted mice.

Inhibition of mitochondrial translation with linezolid might exert antifibrotic effects in a similar manner in macrophages. In line with this, our RNAseq data demonstrate that linezolid can down‐regulate glycolysis in macrophages stimulated with TGFβ (an M2‐polarizing cytokine).^27^ Another study showed that inhibition of mitochondrial translation in inflammatory macrophages leads to their conversion to an immunosuppressive phenotype.^28^ Thus, despite diverging metabolic programs, with M1 macrophages relying predominantly on glycolysis and M2 macrophages relying on OXPHOS and fatty acid oxidation,^29^ both M1‐polarized and M2‐polarized macrophages might require a functional mitochondrial translation, and interfering with mitochondrial translation might induce a transition from either M1‐polarized or M2‐polarized macrophages to resting macrophages.

Accumulating evidence indicates that myofibroblast differentiation is accompanied by metabolic reprogramming, most notably characterized by up‐regulation of glycolysis and glutaminolysis.^8^, ^22^ Furthermore, in SSc and related conditions, mitochondrial dysfunction is closely linked to fibroblast activation; prolonged TGF‐β exposure downregulates the mitochondrial transcription factor TFAM and compromises oxidative phosphorylation to promote fibroblast activation and tissue fibrosis.^6^ Our experiments demonstrate that inhibition of mitochondrial protein synthesis exacerbates mitochondrial dysfunction in SSc fibroblasts, leading to reduced oxygen consumption and a lower NAD^+^/NADH ratio, which ultimately diminishes collagen production. These findings suggest that although mitochondrial TCA cycle and ETC activities are reduced compared with healthy fibroblasts, they remain critically required to support the glycolytic program that sustains collagen production in SSc myofibroblasts. Given that fibroblast activation and excessive collagen biosynthesis rely heavily on glycolysis for both energy and metabolic precursors,^23^ targeting glycolysis has been shown to mitigate fibrotic progression in several organs, including the heart and lungs.^23^, ^26^ In this context, inhibition of mitochondrial translation may represent a novel therapeutic approach to reverse pathologic fibroblast activation by exploiting vulnerabilities in metabolic reprogramming in SSc and other fibrotic disorders.

Treatment with linezolid was well tolerated in our mouse model, and linezolid demonstrated good tolerability in humans when employed for its antibiotic effects.^30^ However, longer‐term administration for their antifibrotic effects in patients with SSc might increase the risk for Gram‐negative infections and thus raise concerns regarding antimicrobial resistance and alterations in gut microbiota.^31^ Inhibition of TGFβ‐induced fibroblast‐to‐myofibroblast transition or reduction on intratissular inflammatory infiltrates might negatively impact physiologic wound healing. Additionally, chronic use of linezolid has been associated with rare but serious adverse events such as interstitial lung disease, as noted in recent safety reviews.^32^ These aspects underscore the need for cautious long‐term evaluation. Future work in additional in vivo models will be required to confirm the efficacy and safety of linezolid in SSc or to evaluate the antifibrotic potential of other nonantibiotic inhibitors of mitochondrial translation such as members from the argyrin family.^3^

## AUTHOR CONTRIBUTIONS

All authors contributed to at least one of the following manuscript preparation roles: conceptualization AND/OR methodology, software, investigation, formal analysis, data curation, visualization, and validation AND drafting or reviewing/editing the final draft. As corresponding author, Dr Matei confirms that all authors have provided the final approval of the version to be published and takes responsibility for the affirmations regarding article submission (eg, not under consideration by another journal), the integrity of the data presented, and the statements regarding compliance with institutional review board/Declaration of Helsinki requirements.

## Supporting information


**Disclosure Form**:

**Data S1** Supporting Information.
