# Supplementary material for: Linezolid‐mediated Prevention of Fibroblast Activation and Tissue Fibrosis via Mitochondrial Translation Inhibition
Source: Arthritis Rheumatol. 2026 Feb 13;78(4):914–27. doi: 10.1002/art.43440 (PMC13054457; doi:10.1002/art.43440)
Supplement: Supplementary file 2 — Data S1 Supporting Information. [file ART-78-914-s001.docx]

**Supplemental Information**

**Linezolid prevents fibroblast activation and ameliorates tissue fibrosis by inhibition of mitochondrial translation**

Xuezhi Hong, MD^1,2,3,4*^, Yanhua Xiao, MD^1,2,3,4*^, Haodong Qi, MMed^1,2^, Shihao Zhu, MMed^1,2^, Tim Filla, PhD^1,2^, Andrea-Hermina Györfi, MD^1,2,5^, Yi-Nan Li, PhD^1,2^, Meilin Xu, MMed^1,2^, Langxian Zhi, MMed^1,2^, Thuong Trinh-Minh PhD^1,2^, Clara Dees, PhD^3,4^, Georg Schett, MD^3,4^, Jörg H.W. Distler, MD^1,2,5^, Alexandru-Emil Matei, MD^1,2,5#^

^1^Department of Rheumatology, University Hospital Düsseldorf, Medical Faculty of Heinrich Heine University, Düsseldorf, Germany.

^2^Hiller Research Center, University Hospital Düsseldorf, Medical Faculty of Heinrich Heine University, Düsseldorf, Germany.

^3^Department of Internal Medicine 3, Rheumatology and Clinical Immunology, Friedrich-Alexander-University Erlangen-Nürnberg (FAU) and University Hospital Erlangen, Erlangen, Germany.

^4^Deutsches Zentrum Immuntherapie (DZI), University Hospital Erlangen, Kussmaulallee 4, 91054, Erlangen, Germany.

^5^Fraunhofer Institute for Translational Medicine and Pharmacology ITMP, and Fraunhofer Cluster of Excellence for Immune Mediated Diseases CIMD, Frankfurt am Main, Germany.

**#** Contributed equally

***Corresponding author:**

Alexandru-Emil Matei, MD; Department of Rheumatology and Hiller Research Center, University Hospital Düsseldorf, Heinrich Heine University, 40225 Düsseldorf, Germany, Email: alexandru-emil.matei@med.uni-duesseldorf.de

**Key words:** fibroblasts, fibrosis, linezolid, mitochondrial translation, systemic sclerosis

**Materials and methods**

**Cell culture**

Fibroblasts were isolated as previously described^1,2^ and cultured in DMEM/F-12 medium (GIBCO, Carlsbad, CA, USA, 31330-038) supplemented with 10% FBS (Thermo Fisher, Carlsbad, CA, USA, 10270-016), 1% penicillin/streptomycin (Life Technologies, Carlsbad, CA, USA, 15140-122), 0.2% amphotericin B (Thermo Fisher, 15290018), and 0.5% L-glutamine (Life Technologies, 255030-024)^1^. Fibroblasts were treated with 10 ng/mL TGFβ (PeproTech, Hamburg, Germany, #100-21C) alone or in combination with 100 μM Linezolid (MedChemExpress, MCE, New Jersey, USA, HY-10394).

Human keratinocytes (PromoCell, Heidelberg, Germany, C-12001) were obtained at passage 2 and cultured according to the manufacturer's instructions. Briefly, cells were grown in Growth Medium 2 (PromoCell, C-20011) and splitted at a 1:8 ratio when they reached 80% confluence. Cells were frozen upon reaching 80–90% confluence. Passage 4 keratinocytes were used for the experiments.

**Quantitative real-time PCR**

Total RNA was extracted using Nucleospin kits (Macherey-Nagel, Düren, Germany) according to the manufacturer’s instructions, then reverse-transcribed into cDNA. Quantitative real-time PCR was performed on the QuantStudio 6 Flex System (Thermo Fisher Scientific). Primer sequences are provided in Table S1. Beta-actin served as the reference gene for normalizing cDNA levels across samples. Relative gene expression was calculated using the comparative Ct (ΔΔCt) method, based on differences in cycle threshold (Ct) values.

**Western blot analysis**

Proteins were resolved using SDS-PAGE and transferred to PVDF membranes. The membranes were incubated overnight at 4°C with primary antibodies targeting collagen I (Abcam, Cambridge, UK, ab138492, 1:2500,) MTCO1 (Abcam, ab14705, 1:1000), SDHA (Abcam, ab14715, 1:2500), and β-Actin (Sigma-Aldrich, A5441, 1:10000). Secondary antibodies conjugated with horseradish peroxidase (HRP) (Dako, Hamburg, Germany, P0447/P0448, 1:5000) were used for detection. Protein visualization was achieved using enhanced chemiluminescence (ECL, GE Healthcare, USA) and imaged on a ChemiDoc MP Imaging System (BioRad, Hercules, CA, USA). β-actin served as a loading control to confirm equal protein amounts. Band intensity quantification was conducted using Image Lab software (version 6.0.0, BioRad).

**Stress fiber staining**

Stress fiber staining was performed as described^3^. Fibroblasts were treated as for the HPG assay. After 7 days, the cells were washed once with PBS, fixed, and permeabilized. Unspecific antibody binding was then blocked by incubation with 2% BSA/PBS for 1 hour. Following blocking, the cells were incubated overnight at 4°C with anti-αSMA antibody (Sigma, A5228) and Rhodamine Phalloidin (Invitrogen, Regensburg, Germany, R415). On next day, cells were stained with secondary antibodies conjugated to Alexa Fluor 488 and counterstained with DAPI for 1 hour. Afterward, the cells were washed twice with PBS and mounted using 100 µL of 50% glycerol in PBS.

**Extracellular matrix (ECM) staining**

The ECM staining was performed as described^4^. Fibroblasts were cultured under different experimental conditions (Vehicle, TGFβ, TGFβ + linezolid) for 5 days. After stimulation, cells were detached and seeded onto a 96-well black-walled imaging plates (Corning, NY, USA, 353219) at a density of 5000 cells per well, followed by stimulation for another 5 days. Presto Blue assays were conducted to normalize for variations in cell number. Subsequently, the plates were rinsed with PBS, and cells were removed using 0.25 M ammonium hydroxide in 25 mM Tris buffer for 15 minutes at 37 °C. The plates, containing the remaining ECM, were washed three times with PBS, fixed in 100% methanol at −20 °C for 30 minutes, and then rinsed three additional times with PBS. The ECM was immunostained with Alexa Fluor 488-conjugated monoclonal mouse anti-Fibronectin antibodies (eBiosciences, San Diego, CA, USA, 53-9869-82), monoclonal rabbit anti-Collagen type 1 antibodies (Abcam, ab138492). CellInsight CX5 was used for image capture and analysis.

**Gel contraction assay**

To assess fibroblast contractility, a collagen gel contraction assay was established based on the protocol of the three-dimensional SSc skin equivalent, with the keratinocyte and macrophage seeding step omitted. Dermal fibroblasts (0.2 × 10⁶ cells) were suspended in 2.5 mg/mL rat tail collagen I and seeded into 12-well plates (500 μL/well). After gel polymerization, the models were cultured in fibroblast medium and treated with either TGF-β1 (10 ng/mL) or TGF-β1 and linezolid (100 μM) for 7 days. Gel areas were documented at the end of the experiment and quantified using ImageJ.

**Cell viability assay**

Fibroblasts were seeded in 96 well plates at a density of 1200 cells per well. After 24 hours of serum deprivation, cells were stimulated with 12.5µM, 25µM, 50µM, 100µM, 200µM Linezolid or vehicle for 7 days. The medium was replaced with 90 µl of fibroblast medium and 10 µl of Cell Counting Kit-8 (CCK-8, abcam, ab228554) solution per well. The plates were then incubated at 37℃ for 1h. The optical density (OD) value of each well was measured at 450 nm with a microplate reader (Tecan Infinite M200 Pro, Germany).

**Bioinformatic analysis**

RNA was isolated as described^5^ and sent to Novogene (Cambridge, UK, or Munich, Germany) for RNA sequencing using the Illumina NovaSeq system. Principal component analysis (PCA) was used to compare the overall differences in gene expression profiles between different experimental conditions in the murine cGvHD model. Differentially expressed genes (DEGs) were identified by edgeR based on the following thresholds: False Discovery Rate (FDR) < 0.2 and |fold change| > 1.5 for fibroblasts, FDR < 0.05 and |fold change| > 1.5 for the murine cGvHD model, FDR < 0.2 and |fold change| > 1.5 for the PCSS model, and FDR < 0.05 and |fold change| > 1.5 for macrophages. For enrichment analyses, significance thresholds were p-value < 0.05 for fibroblasts and macrophages and adjusted p-value < 0.05 for the murine cGvHD and PCSS models. Gene expression data from the skin of SSc patients were obtained from GSE59787 and were re-analyzed to define SSc_DEGs with a p-adjusted value cut-off of ≤ 0.05 and a |log_2_ fold change| cut-off of ≥ 1, as previously described^6^. Gene expression data from the skin of sclGvHD were re-analyzed from our previous publication^3^ to define patient sclGvHD_DEGs with an adjusted P-value < 0.05 and |fold change| > 1.5. Human genes were converted to their mouse orthologs using the Mouse Genome Informatics (MGI) database. The analysis was performed in R (version 4.2.1).

**Scoring of clinical manifestations of murine sclGvHD**

Mice were monitored daily to assess disease severity based on skin lesions, weight loss, and mobility impairment. Clinical scoring was performed according to established criteria^7^, evaluating coat condition (0-4), skin involvement (0-4), percentage of weight change (0-4), posture (0-3), and activity level (0-3), with a maximum possible score of 18.

**Hydroxyproline assay**

Collagen content in the skin and lungs was measured using the hydroxyproline assay^8^. Skin biopsies (3 mm) were taken from the dorsal skin, and lung samples from the middle lobe of the right lung. Samples were digested in 6 M HCl at 120 °C for 3 hours or overnight, then neutralized with 6 M NaOH (pH 7–8). After adding 0.06 M chloramine T and incubating at room temperature for 20 minutes, 3.15 M perchloric acid, 2-methoxyethanol, and p-dimethylaminobenzaldehyde were added. The mixture was incubated at 60 °C for another 20 minutes, and absorbance was measured at 557 nm using a SpectraMax 190 microplate spectrophotometer (Molecular Devices, Sunnyvale, CA, USA).

**Histological analysis and immunohistochemistry staining**

Skin samples were obtained from the upper back of each mouse, fixed in 4% formalin, and embedded in paraffin. Sections were stained with trichrome, and dermal thickness was measured at four different sites per slide^7^. For lung analysis, the left lung lobe was collected, fixed, embedded in paraffin, and sectioned for trichrome staining. Pulmonary fibrosis was assessed using the Ashcroft score^9^. Colon samples were fixed in formalin, embedded in paraffin, sectioned, and stained with hematoxylin and eosin. The inflamed area was quantified for each mouse. Skin equivalents were fixed, embedded in paraffin, sectioned, and stained with trichrome. Dermal thickness was quantified following established protocols. Skin and lung myofibroblasts from murine cGvHD, as well as myofibroblasts from skin equivalents, were identified by immunohistochemical staining using a monoclonal anti-α-smooth muscle actin (αSMA) antibody (Sigma Aldrich, A5228)^10^. The number of αSMA-positive myofibroblasts was determined by counting across four different areas per slide.

**Immunofluorescence staining**

Paraffin-embedded sections from murine skin and PCSS were stained with primary antibodies against CD45 (Abcam, ab10558), CD3 (Abcam, ab11089), B220 (Thermo Fisher, 14-0452-82), CD68 (Proteintech, 25747-1-AP), P4Hβ (R&D, AF4236) and αSMA. On the following day, slides were incubated with Alexa Fluor 555- or 647-conjugated secondary antibodies and counterstained with DAPI for 1 hour. Fluorescence images were acquired using a ZEISS Axio Observer 7 microscope.

**Seahorse assay**

Human dermal fibroblasts were seeded at a density of 4 × 10⁴ cells per well into Seahorse XF96 microplates (Agilent, Santa Clara, CA, USA, 103794-100), which had been pre-coated overnight with 0.5 mg/ml type I collagen solution derived from rat tails (Sigma-Aldrich). Fibroblasts were then treated sequentially with 1.5 μM oligomycin, 1.0 μM carbonyl cyanide p-trifluoromethoxyphenylhydrazone (FCCP), and 0.5 μM rotenone/antimycin A (all from Agilent, USA, 103015-100). The oxygen consumption rate (OCR) was measured at various time points using an XF-96 Extracellular Flux Analyzer (Agilent, Santa Clara, CA, USA) and processed with Wave software (version 2.6.0, Agilent)^11^.

**NAD⁺/NADH ratio measurement**

The NAD⁺/NADH ratio was measured in fibroblasts treated with Linezolid for 7 days using the NAD/NADH-Glo™ Assay (Promega, Madison, USA, G9071), following the manufacturer's instructions.

**Statistics**

Data are presented as mean ± standard deviation. A paired t-test was used for comparisons between two groups, while one-way ANOVA was applied for analyses involving more than two groups. Tukey’s test was performed for post hoc comparisons following ANOVA. Two-way ANOVA followed by Dunnett’s post-hoc test was performed to analyze clinical scores and weight changes. Statistical significance was set at p < 0.05. P-values are reported as follows: 0.05 ≥ p > 0.01 as *, 0.01 ≥ p > 0.001 as **, and p < 0.001 as ***.

**Ethical approval**

Informed consent was obtained from all donors prior to their inclusion in the study. Procedures involving fibroblast isolation, iPSC reprogramming, and subsequent iPSC-based experiments were conducted in accordance with ethical guidelines and approved by the ethics committees of the University of Erlangen-Nürnberg and the University of Düsseldorf. The corresponding approval numbers are 21-485-Bp, 98_18 B, and 30_19B (Erlangen), as well as 2022-2158 (Düsseldorf).


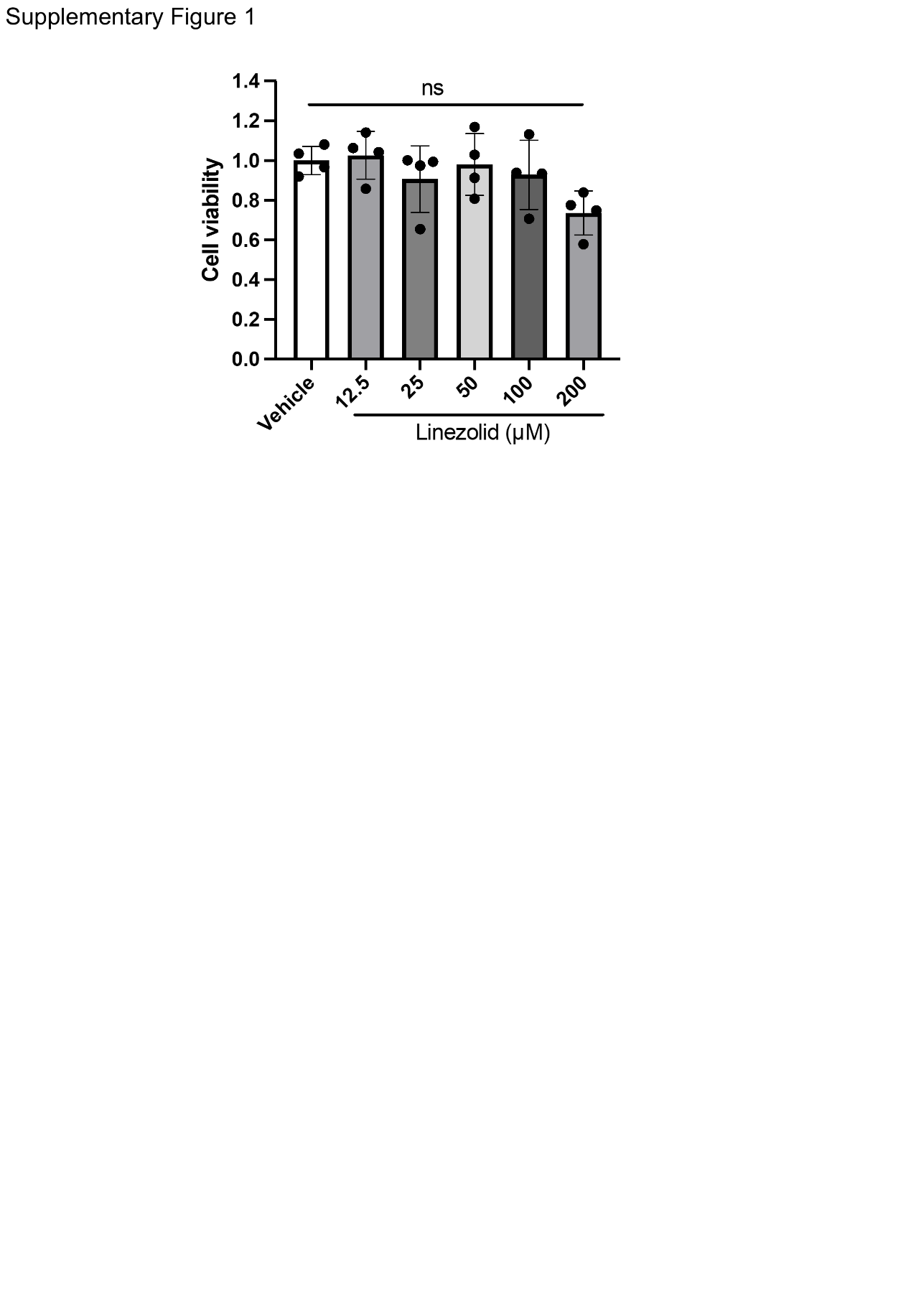
**Supplementary Figure 1. Linezolid does not exert significant toxic effects on cultured human fibroblasts in concentrations used for evaluation of antifibrotic effects.**

Fibroblasts were treated with increasing concentrations of linezolid for 7 days. Cell viability was measured by CCK-8 assay. Data are normalized to vehicle and shown as mean ± SD (n = 4).


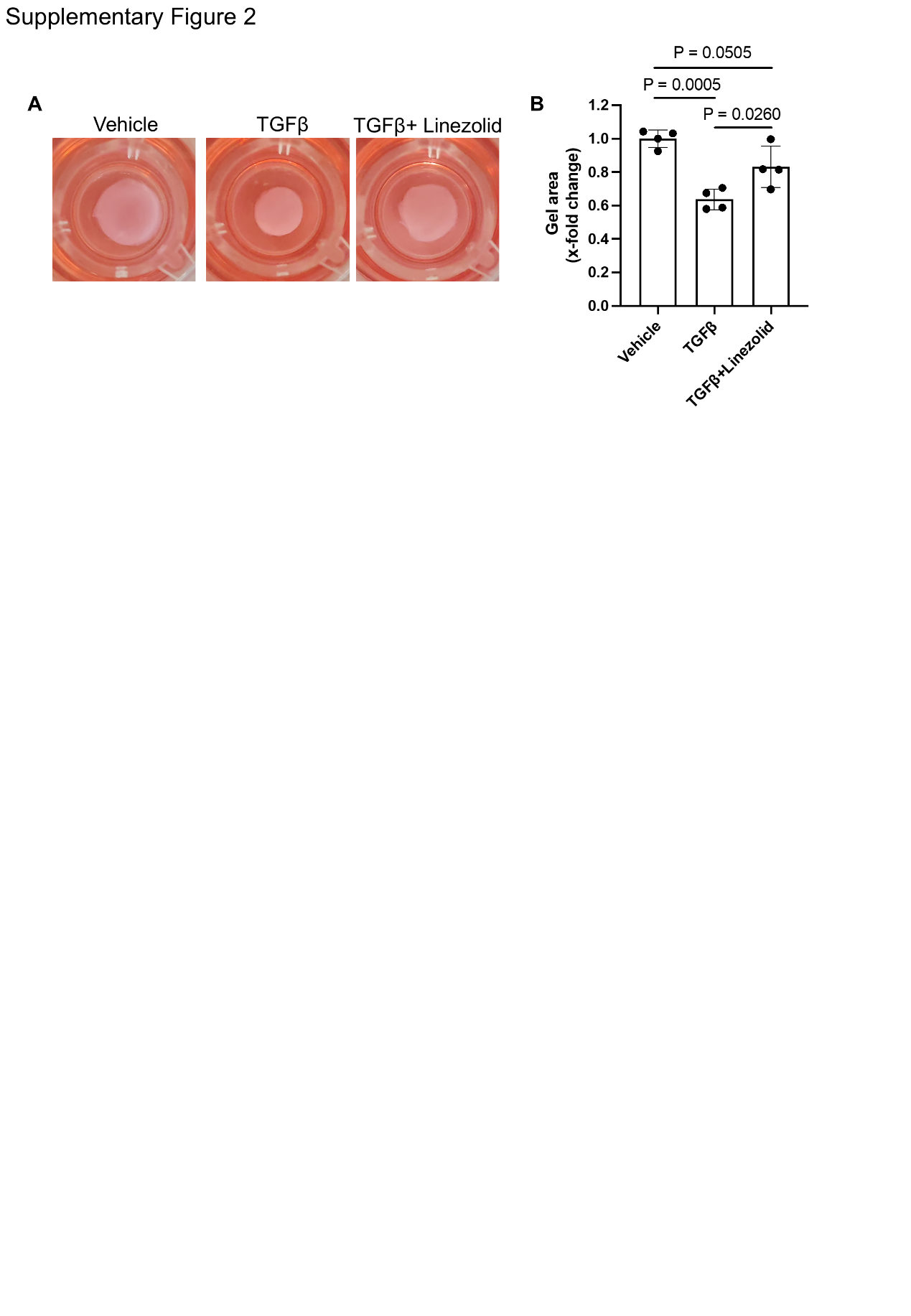


**Supplementary Figure 2. Linezolid reduces TGFβ-induced collagen gel contraction.**

**A**. Representative images of collagen gels containing normal human dermal fibroblasts after treatment with vehicle, TGF-β (10 ng/mL), or TGF-β plus linezolid (100 μM) for 7 days. **B**. Quantification of gel area relative to vehicle-treated controls. Data are presented as mean ± SD (n = 4). Statistical analysis was performed using one-way ANOVA with Tukey’s post hoc test.


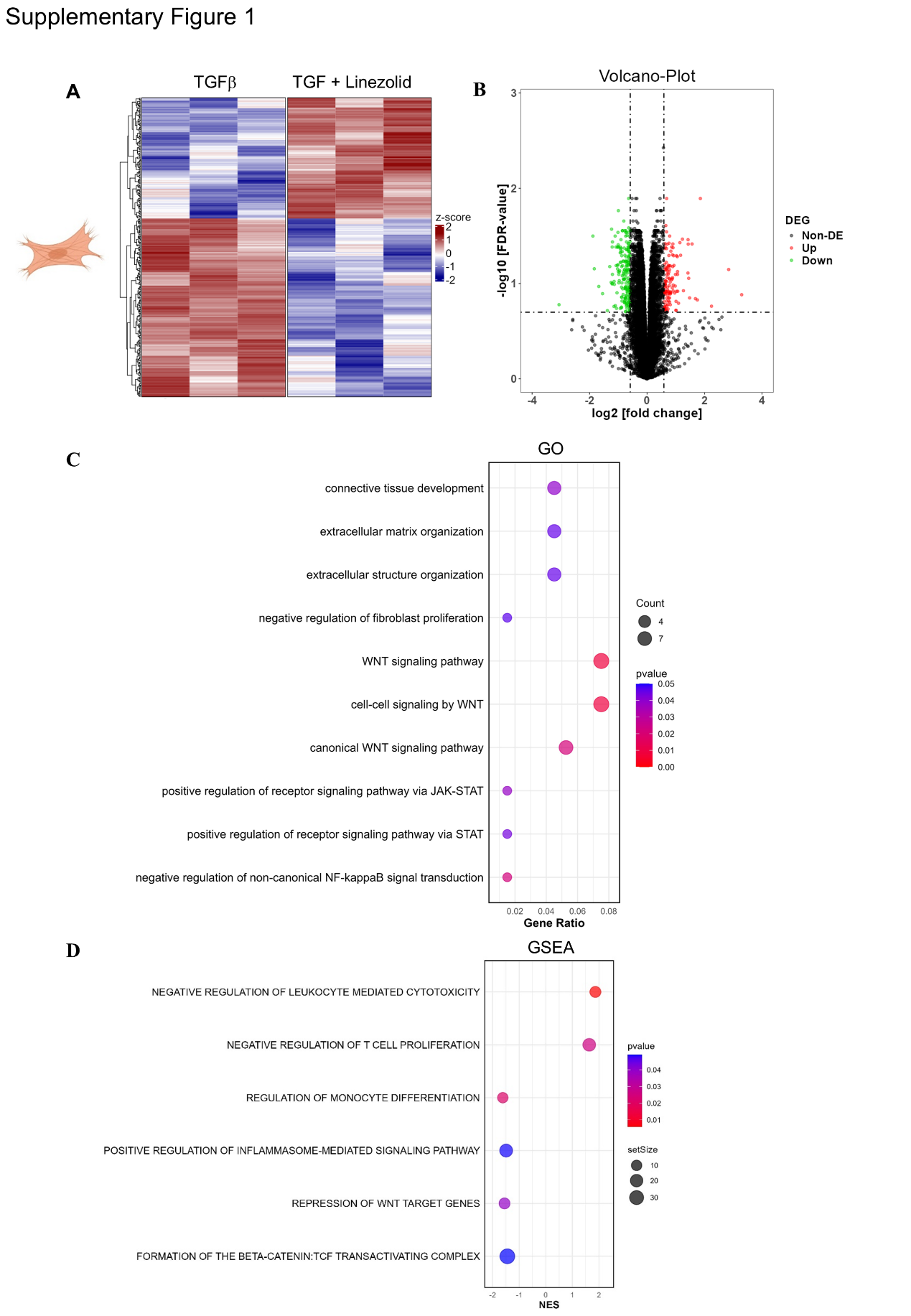


**Supplementary Figure 3. Linezolid reverts profibrotic and proinflammatory gene expression programs in cultured human fibroblasts exposed to TGFβ.**

**A-B.** Heatmap (A) and volcano plot (B) illustrating DEGs in fibroblasts treated with linezolid + TGFβ (n = 3) vs. TGFβ alone (n = 3). **C-D.** Bubble plots highlighting significantly enriched biological processes related to inflammation and fibrosis in human fibroblasts based on Gene Ontology (GO) analysis (C) and Gene Set Enrichment Analysis (GSEA) (D). In both plots, bubble color represents p-values, while bubble size indicates the number of DEGs associated with each GO term (C) or the number of genes linked to each GSEA pathway (D). DEG: differentially expressed genes; JAK: janus kinases; STAT: signal transducer and activator of transcription.


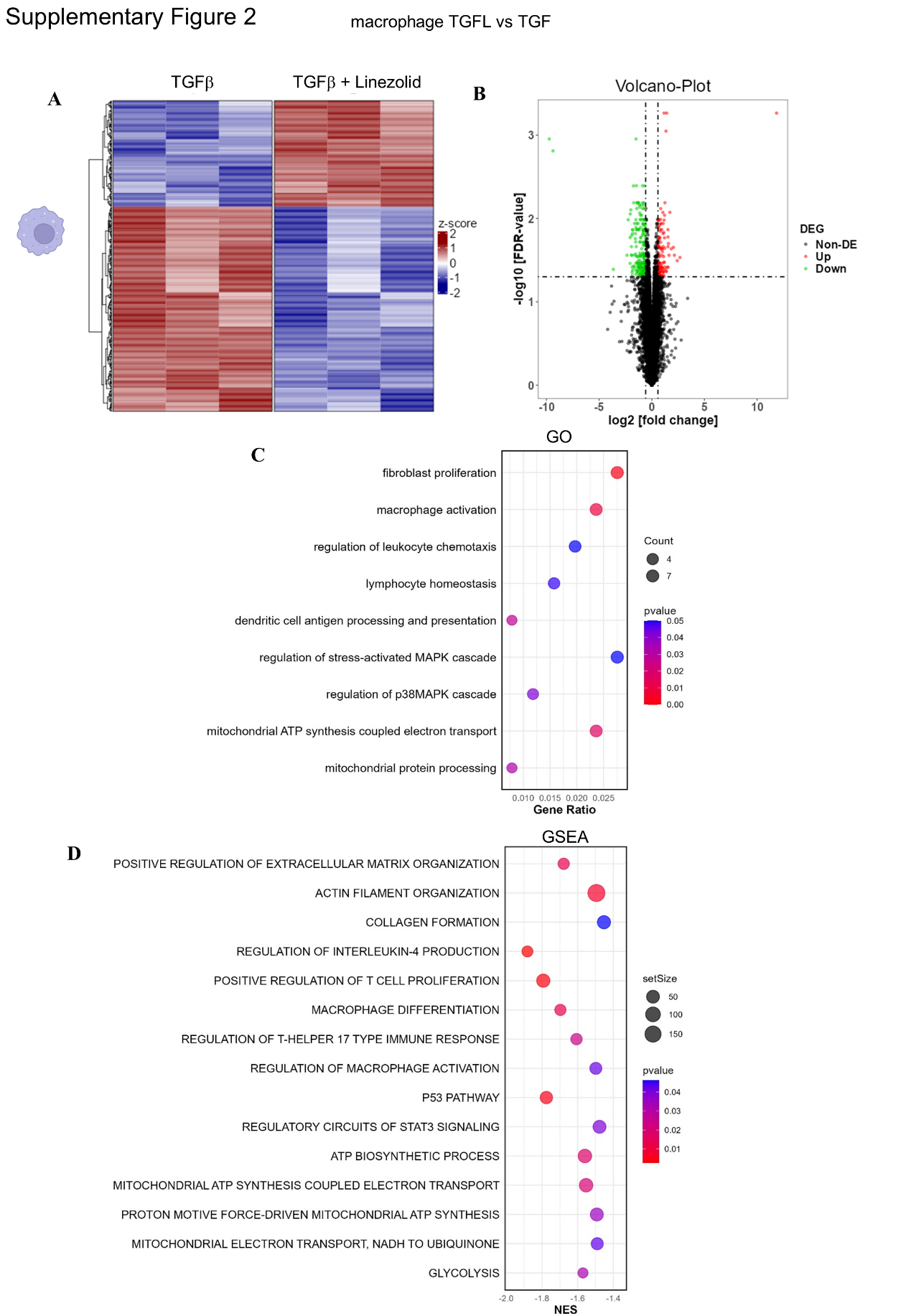


**Supplementary Figure 4. Linezolid reverts gene expression programs related to inflammation and fibroblast activation in human macrophages under M2-polarizing conditions.**

**A-B.** Heatmap (A) and volcano plot (B) illustrating DEGs in macrophages treated with Linezolid + TGFβ (n = 3) vs. TGFβ alone (n = 3). **C-D.** Bubble plots highlighting significantly enriched biological processes associated with inflammation and fibrosis in Linezolid-treated macrophages, based on Gene Ontology (GO) pathway analysis (C) and Gene Set Enrichment Analysis (GSEA) (D). DEGs: Differentially Expressed Genes; TGFβ: transforming growth factor-β; MAPK: Mitogen-Activated Protein Kinase.


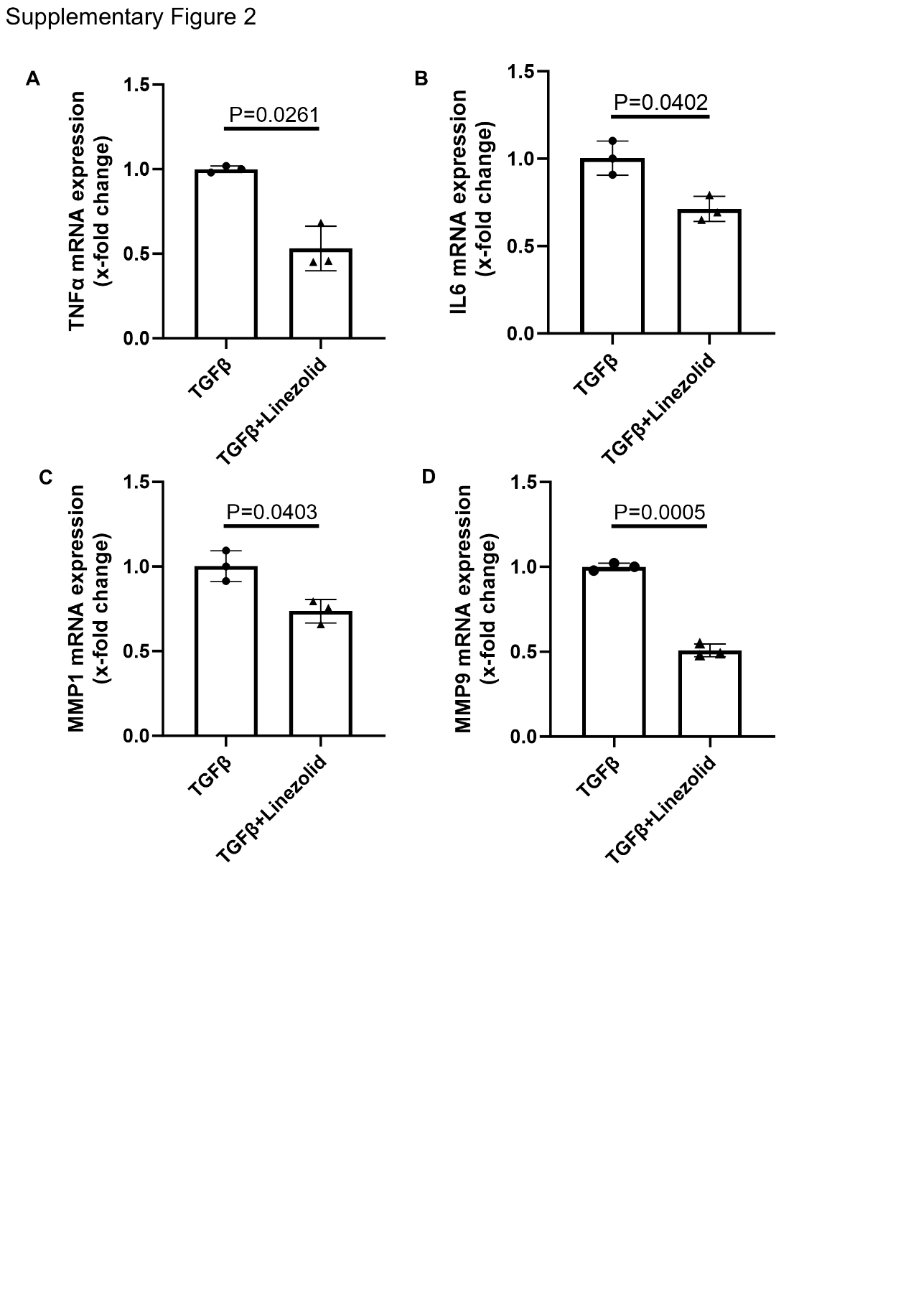
**Supplementary Figure 5. Linezolid reduces the expression of pro-inflammatory and matrix remodeling genes in the three-dimensional SSc skin equivalents.**

**A–D**. mRNA levels of *TNFα* (A), *IL-6* (B), *MMP1* (C) and *MMP9* (D) measured by qPCR in the three-dimensional SSc skin equivalents following stimulation with TGFβ, with or without linezolid treatment. Data are shown as mean ± SD from three independent biological replicates (n = 3). Statistical significance was assessed using paired t-tests. TNFα: tumor necrosis factor alpha; IL-6: interleukin-6; MMP1: matrix metallopeptidase 1; MMP9: matrix metallopeptidase 9.

**
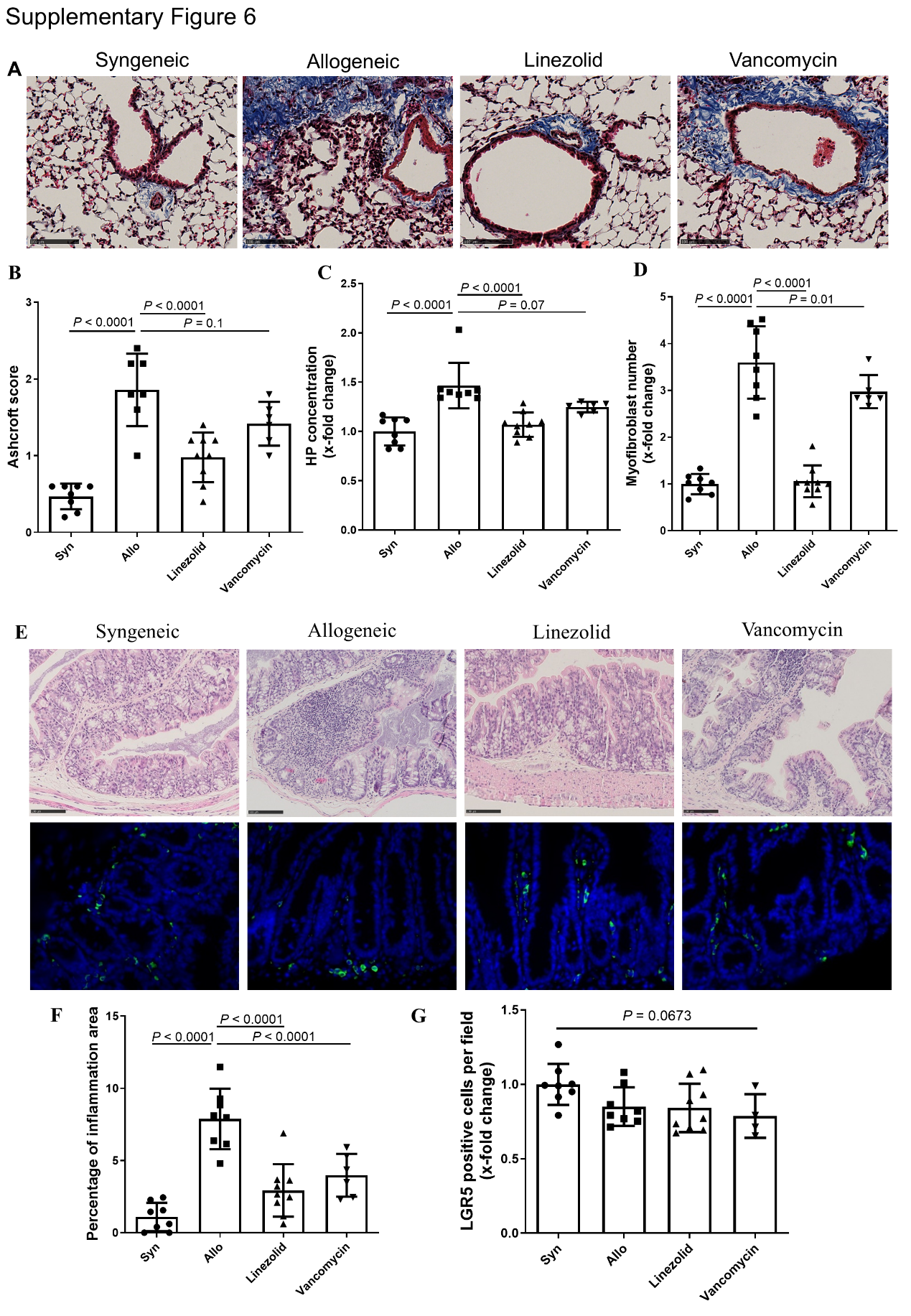
**

**Supplementary Figure 6. Linezolid ameliorates lung fibrosis and intestinal inflammation in murine sclGvHD.**

**A.** Representative trichrome-stained lung sections from sclGvHD mice (scale bar = 250 µm). **B-D.** Quantification of pulmonary fibrosis, assessed by the Ashcroft score (B), hydroxyproline content (C), and myofibroblast counts (D) in the lungs of sclGvHD mice. **E-F.** Representative HE-stained images of colon (scale bar = 100 µm) and LGR5 immunofluorescence staining of the small intestine at 400× magnification. **F.** Quantification of the inflammatory area in the colon. **G.** Quantification of LGR5-positive stem cells in the small intestine. Data are presented as mean ± SD, with n = 6-9 independent biological samples per group. *P*-values determined by one-Way ANOVA with Tukey’s test. 0.05>*P*>0.01*, 0.01>*P*>0.001**, *P*<0.001***. sclGvHD: sclerodermatous chronic graft-versus-host disease; HE: Hematoxylin & Eosin staining; LGR5: leucine-rich repeat-containing G protein coupled receptor 5.


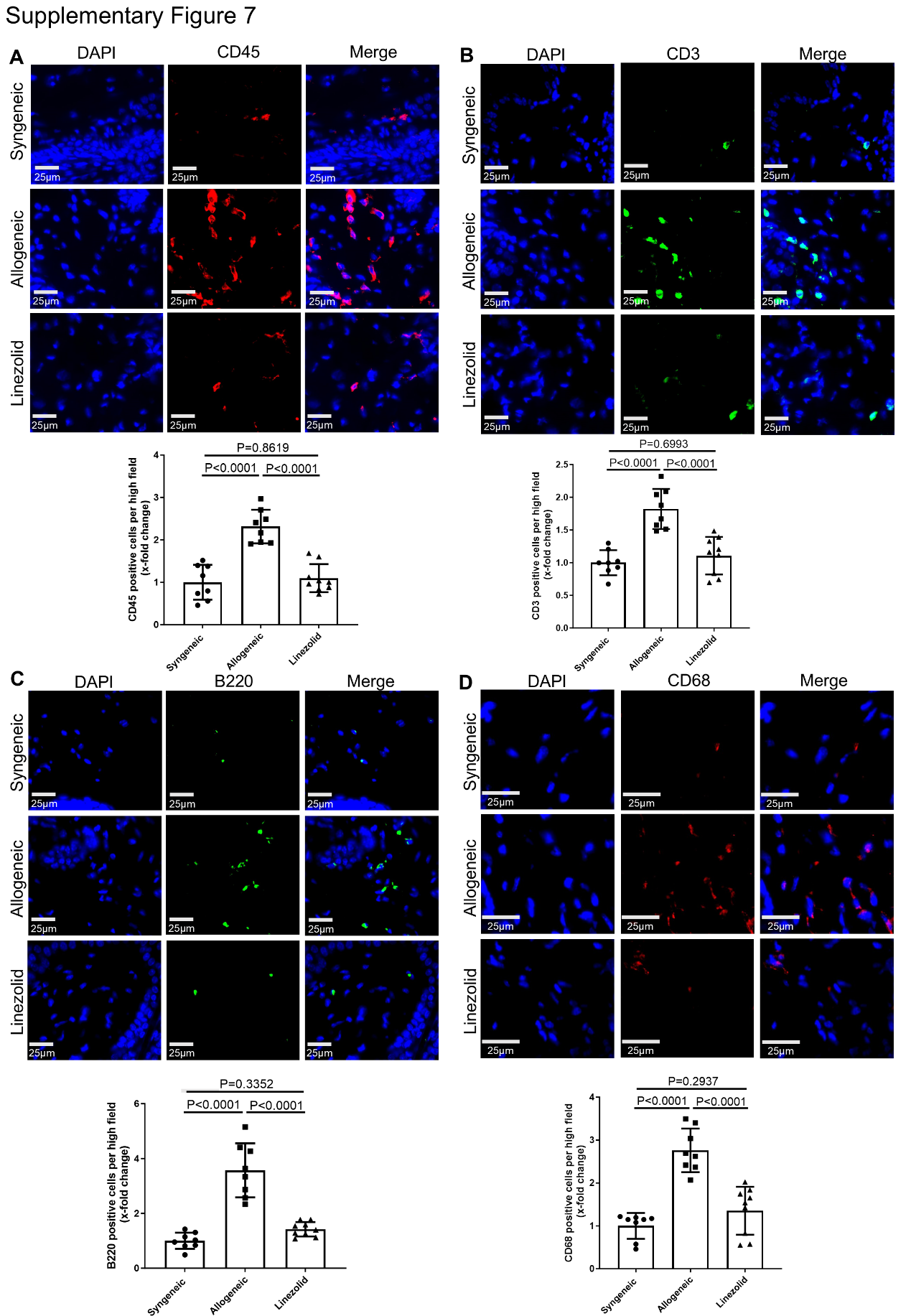


**Supplementary Figure 7. Linezolid reduces immune cell infiltration in the skin of sclGvHD mice.**

**A-D**. Representative immunofluorescence images of skin sections stained for CD45 (A), CD3 (B), B220 (C), and CD68 (D) in syngeneic controls, allogeneic sclGvHD mice, and linezolid-treated sclGvHD mice (Scale bar = 25 μm). Quantification of marker-positive cells per high-power field is included (n = 8–9). Statistical analysis was performed using one-way ANOVA followed by Tukey’s multiple comparisons test. sclGvHD: sclerodermatous chronic graft-versus-host disease.

**
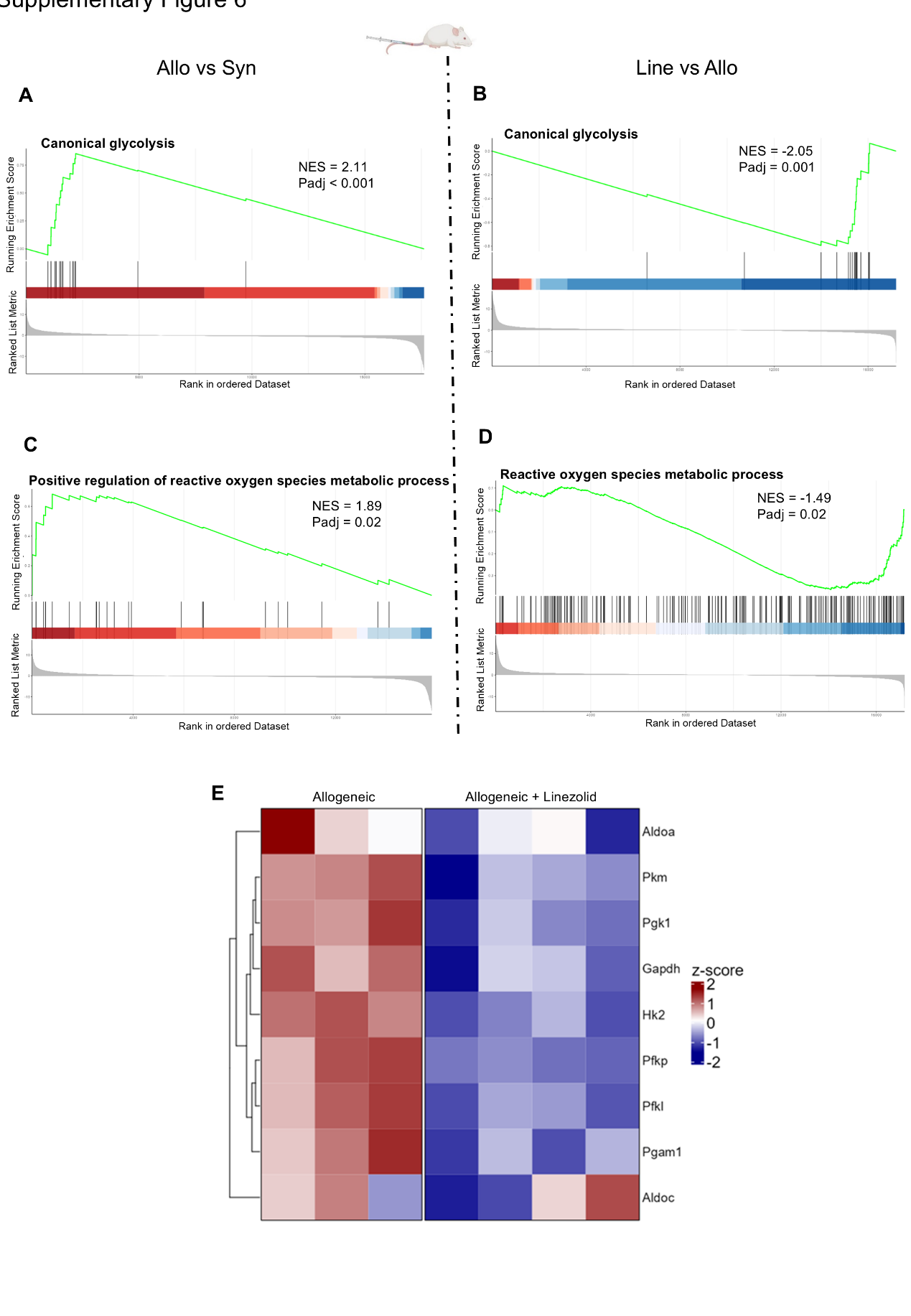
**

**Supplementary Figure 8. Linezolid inhibits glycolysis and ROS metabolic process in murine sclGvHD.**

**A-D.** Gene set enrichment analysis (GSEA) of the pathway “canonical gylcolysis” (A, B) or “positive regulation of reactive oxygen species metabolic process” (C, D) in allogeneically vs. syngeneically transplanted mice (A, C) or in allogeneically transplanted mice treated with linezolid vs. vehicle (B, D). **E.** Heatmap illustrating the changes expression profile of genes involved in the glycolysis pathway in sclGvHD mice upon treatment with linezolid. ROS: reactive oxygen species; NES: normalized enrichment scores; sclGvHD: sclerodermatous chronic graft-versus-host disease.

**
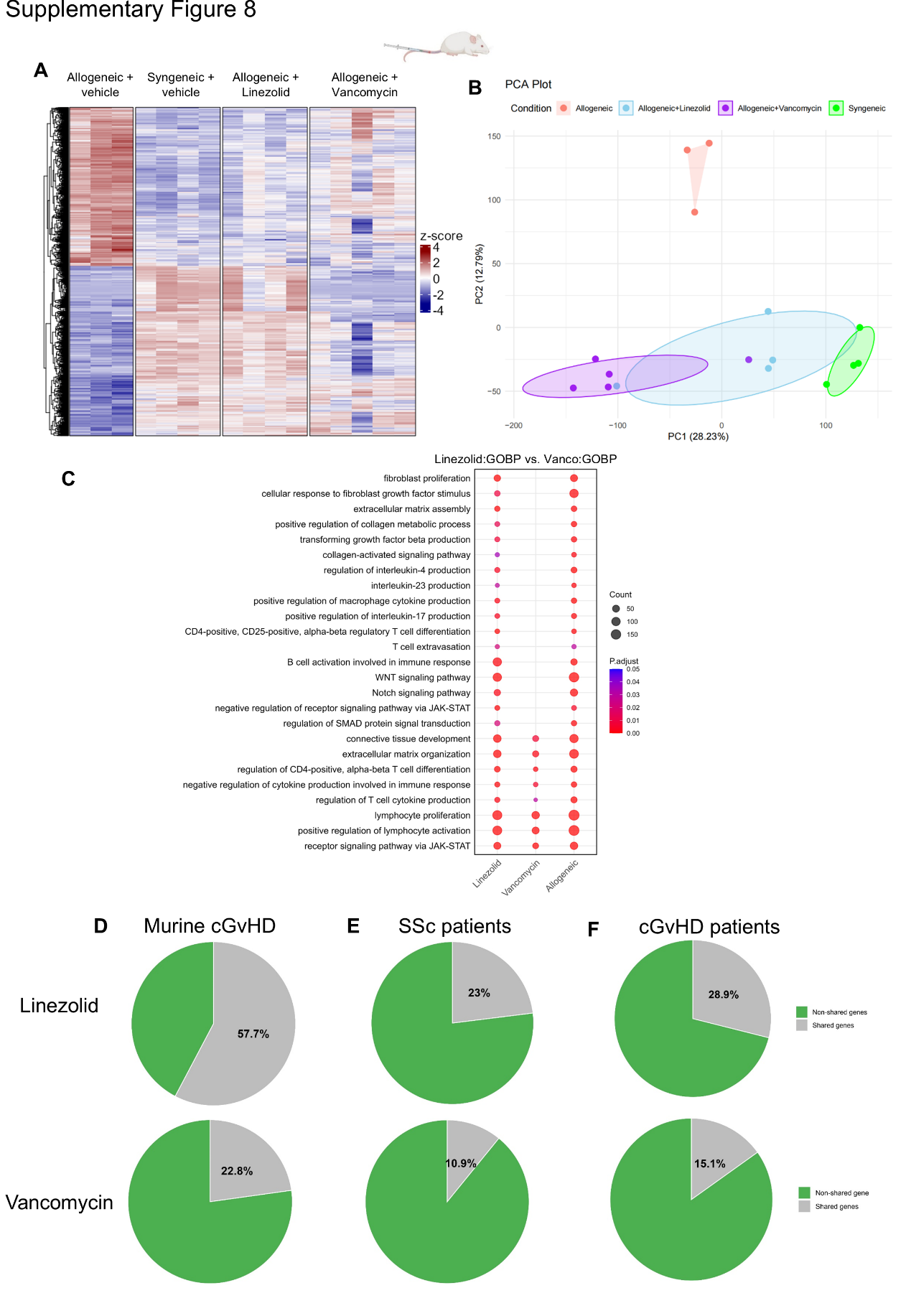
**

**Supplementary Figure 9. Treatment with linezolid corrects the aberrant gene expression program from sclGvHD skin to a higher extent than vancomycin.**

**A.** Heatmap displaying DEGs in syngeneic mice (n=4) and allogeneic mice treated with vehicle (n=3), linezolid (n=4), or vancomycin (n=5). **B.** Principal component analysis (PCA) illustrating the proximity in gene expression profile between syngeneically transplanted mice and allogeneically transplanted mice treated with linezolid, vancomycin or vehicle. **C.** Bubble plot highlighting selected Gene Ontology (GO) pathways related to inflammation or fibrosis in allogeneic mice treated with linezolid, vancomycin, or vehicle. **D-F.** Percentage of overlap between Line-DEGs (DEGs in the skin of allo-GvHD treated with linezolid vs. vehicle) or Vanco-DEGs (DEGs in the skin of allo-GvHD treated with vancomycin vs. vehicle) with murine allo-DEGs (DEGs in the skin allogeneically vs. Syngeneically transplanted mice) (D), with SSc-DEGs (DEGs in the skin of SSc patients vs. controls) (E) and with human sclGvHD-DEGs (DEGs in the skin of sclGvHD patients vs. controls) (F). The percentages were calculated based on the number of overlapping DEGs relative to the total number of murine allo-DEGs (D), of SSc-DEGs (E) or of sclGvHD-DEGs (F). Gray indicates shared DEGs, and green represents non-overlapping DEGs. DEG: differentially expressed genes; GO: Gene Ontology; cGvHD: chronic graft-versus-host disease; SSc: systemic sclerosis; JAK: janus kinases; STAT: signal transducer and activator of transcription; SMAD: Suppressor of Mothers Against Decapentaplegic.


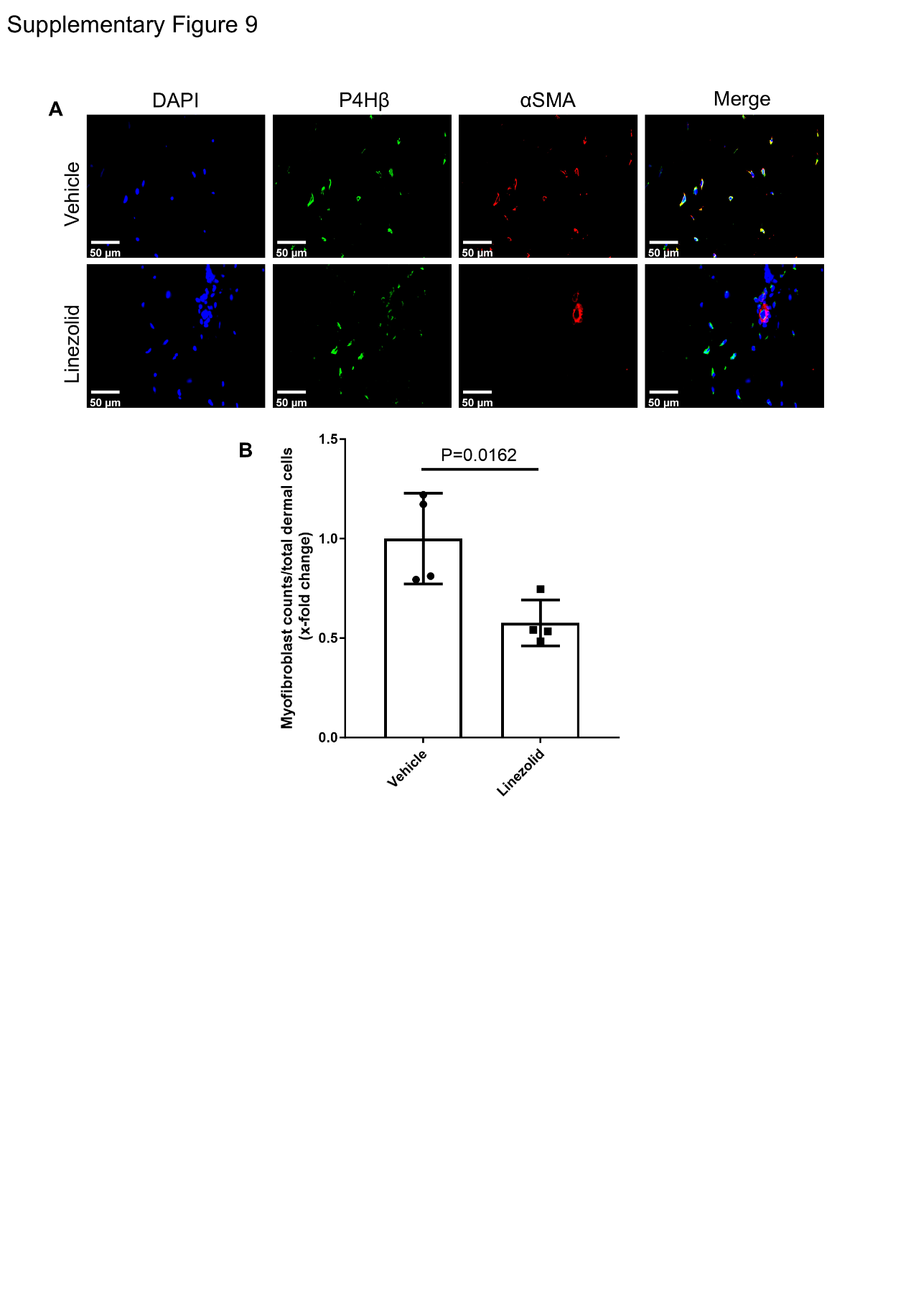


**Supplementary Figure 10. Linezolid reduces fibroblast activation in ex vivo human skin biopsies.**

**A**. Representative immunofluorescence images of ex vivo treated skin biopsies stained for αSMA (red), P4Hβ (green), and DAPI (blue) (Scale bar = 50μm). **B**. Quantification of αSMA⁺/P4Hβ⁺ double-positive cells per high-power field (n = 4). Data were analyzed using unpaired two-tailed t-test.

**
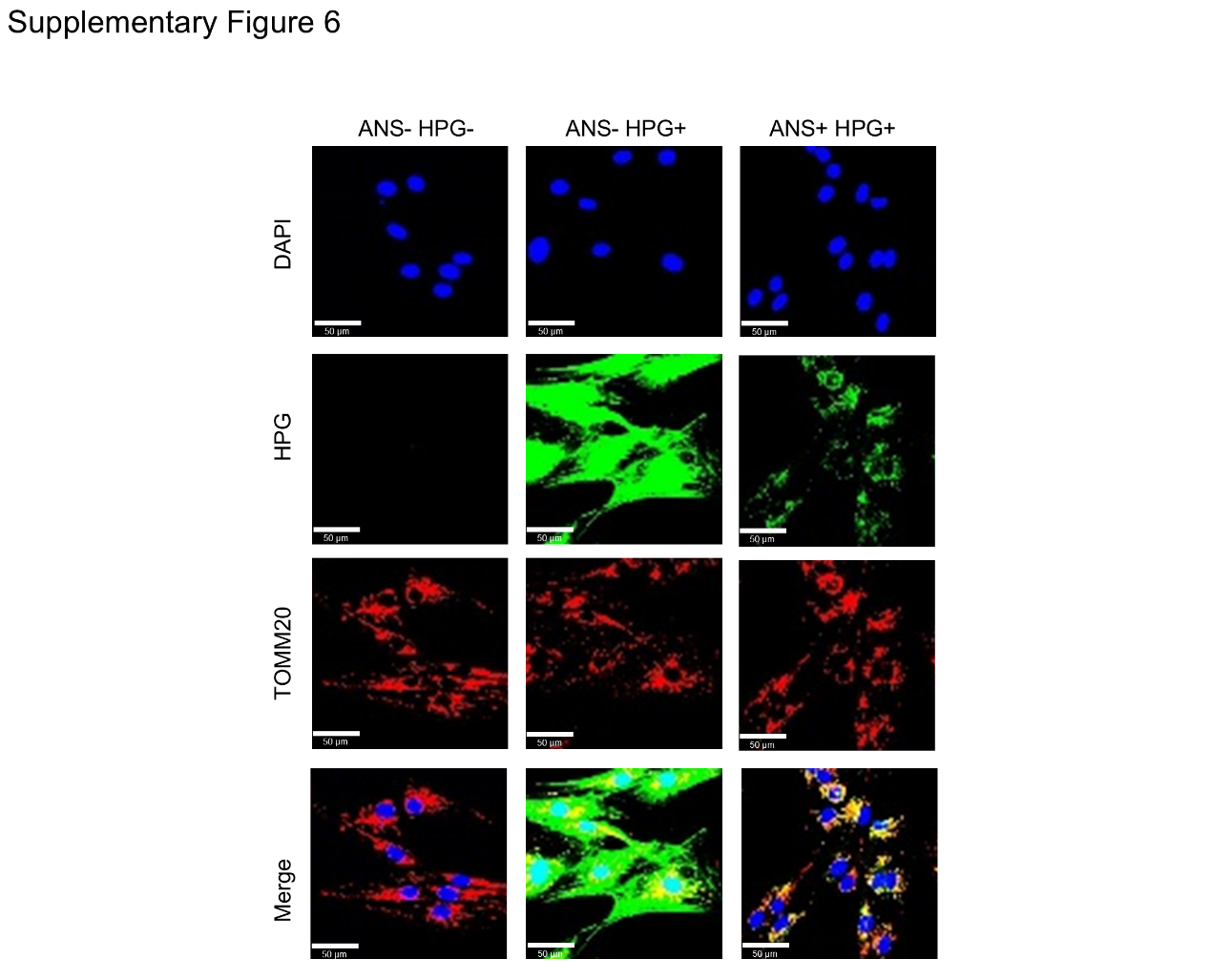
**

**Supplementary Figure 11. Selective incorporation of L-homopropargylgycine in nascent mitochondrial proteins in human fibroblasts upon anisomycin treatment.**

Representative images of L-homopropargylgycine (HPG, green) and the mitochondrial protein TOMM20 (red), showing incorporation of HPG in both mitochondrial and cytosolic nascent proteins in the absence of anisomycin (ANS), and selective incorporation of HPG in mitochondrial nascent proteins in the presence of ANS in cultured human fibroblasts. Scale bars = 50 µm.

**
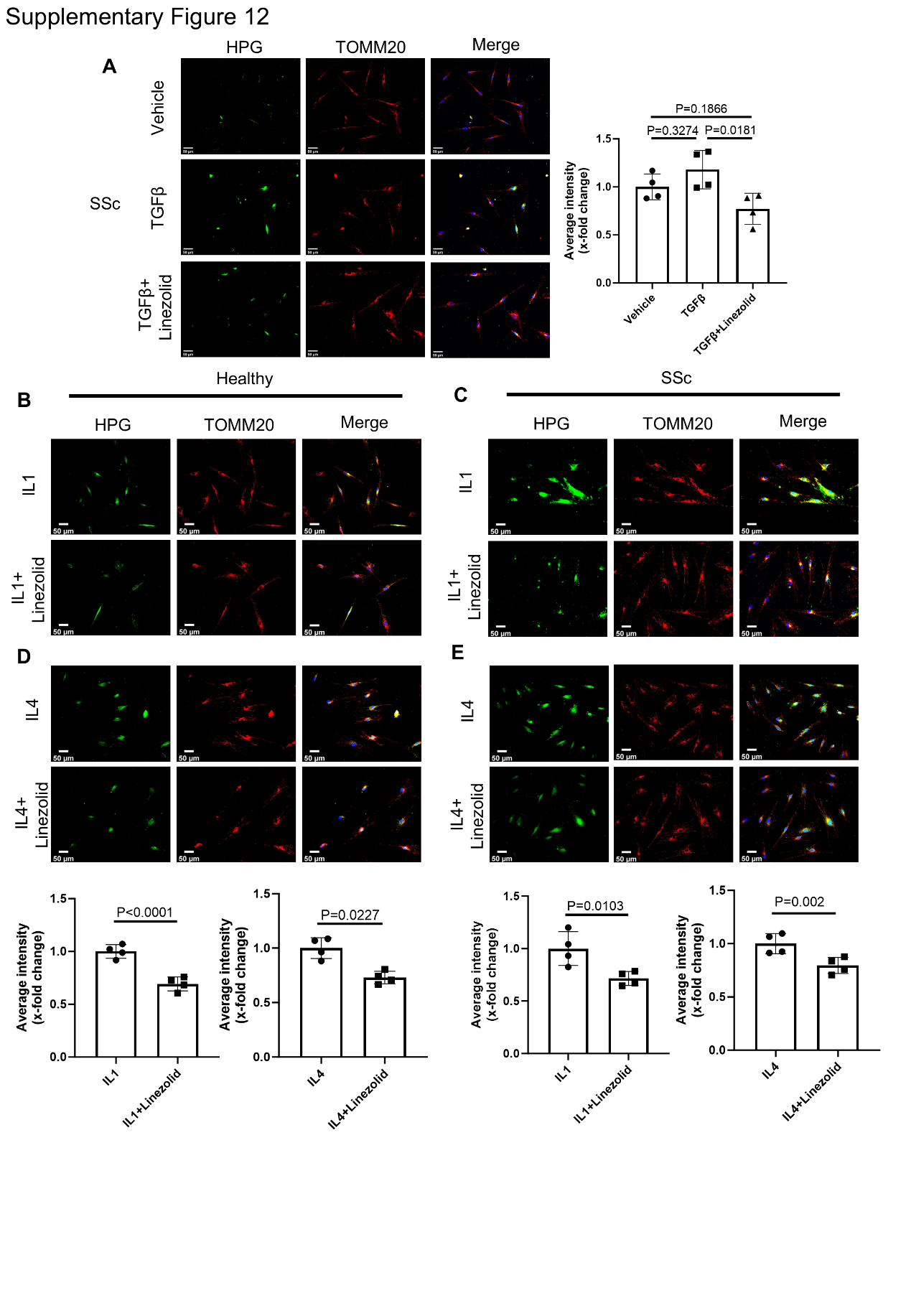
**

**Supplementary Figure 12. Linezolid inhibits mitochondrial protein synthesis in healthy and SSc fibroblasts exposed to TGFβ, IL-1β or IL-4.**

**A-E**. Representative immunofluorescence images of fibroblasts from SSc patients (A) and from healthy controls (B, D) or SSc patients (C, E) stimulated with TGFβ (A), IL-1β (B, C), or IL-4 (D, E), with or without linezolid for 7 days. Cells were stained with HPG (green), TOMM20 (red), and DAPI (blue) (n = 4). Quantification of average intensity of HPG, showing incorporation of HPG in mitochondrial nascent proteins were included. Data were analyzed using a paired t-test or one-way ANOVA followed by Tukey’s post-hoc test. Scale bar: 50 μm.

**Table S1. List of primers used**

| Human ACTB | Fwd | CCT CGC CTT TGC CGA TCC |
| --- | --- | --- |
|  | Rev | GAG CGC GGC GAT ATC ATC A |
| Human COL1A1 | Fwd | ACG AAC ACA TCC CAC CAA TC |
|  | Rev | ATG GTA CCT GAG GCC GTT C |
| Human ACTA2 | Fwd | AAG AGG AAT CCT GAC CCT GAA |
|  | Rev | TGG TGA TGA TGC CAT GTT CT |
| Human FN1 | Fwd | TTC TAA GAT TTG GTT TGG GAT CAA T |
|  | Rev | TCT TGG TTG GCT GCA ATG C |
| Human TNFα | Fwd | TAG CAA ACC CTC AAG CTG |
|  | Rev | GGA GTA GAT GAG GTA CAG G |
| Human IL6 | Fwd | GGA TTC AAT GAG GAG ACT TGC |
|  | Rev | CTC TGG CTT GTT CCT CAC TAC |
| Human MMP1 | Fwd | AAG ACA AAG GCA AGT TGA AAA |
|  | Rev | GTT TCC CAG TCA CTT TCA GC |
| Human MMP9 | Fwd | GGA GCA CGG AGA CGG GTA T |
|  | Rev | CTT GCC CAG GGA CCA CAA CTC |

**References**

1. Liang M, Dickel N, Gyorfi AH, SafakTumerdem B, Li YN, Rigau AR, et al. Attenuation of fibroblast activation and fibrosis by adropin in systemic sclerosis. Sci Transl Med. 2024;16(740):eadd6570.

2. Zhou X, Trinh-Minh T, Matei AE, Gyorfi AH, Hong X, Bergmann C, et al. Amelioration of Fibrotic Remodeling of Human 3-Dimensional Full-Thickness Skin by Transglutamase 2 Inhibition. Arthritis Rheumatol. 2023;75(9):1619-27.

3. Zhang Y, Shen L, Dreissigacker K, Zhu H, Trinh-Minh T, Meng X, et al. Targeting of canonical WNT signaling ameliorates experimental sclerodermatous chronic graft-versus-host disease. Blood. 2021;137(17):2403-16.

4. Zehender A, Li YN, Lin NY, Stefanica A, Nuchel J, Chen CW, et al. TGFbeta promotes fibrosis by MYST1-dependent epigenetic regulation of autophagy. Nat Commun. 2021;12(1):4404.

5. Dees C, Potter S, Zhang Y, Bergmann C, Zhou X, Luber M, et al. TGF-beta-induced epigenetic deregulation of SOCS3 facilitates STAT3 signaling to promote fibrosis. J Clin Invest. 2020;130(5):2347-63.

6. Gronberg C, Rattik S, Tran-Manh C, Zhou X, Rius Rigau A, Li YN, et al. Combined inhibition of IL-1, IL-33 and IL-36 signalling by targeting IL1RAP ameliorates skin and lung fibrosis in preclinical models of systemic sclerosis. Ann Rheum Dis. 2024;83(9):1156-68.

7. Hong X, Xiao Y, Xu L, Shen L, Neelagar R, Devakumar V, et al. Combination therapies of porcupine inhibition with ruxolitinib, ibrutinib or belumosudil in murine sclerodermatous GvHD. Blood Adv. 2025.

8. Kramer M, Dees C, Huang J, Schlottmann I, Palumbo-Zerr K, Zerr P, et al. Inhibition of H3K27 histone trimethylation activates fibroblasts and induces fibrosis. Ann Rheum Dis. 2013;72(4):614-20.

9. Hubner RH, Gitter W, El Mokhtari NE, Mathiak M, Both M, Bolte H, et al. Standardized quantification of pulmonary fibrosis in histological samples. Biotechniques. 2008;44(4):507-11, 14-7.

10. Huang J, Beyer C, Palumbo-Zerr K, Zhang Y, Ramming A, Distler A, et al. Nintedanib inhibits fibroblast activation and ameliorates fibrosis in preclinical models of systemic sclerosis. Ann Rheum Dis. 2016;75(5):883-90.

11. Zhou X, Trinh-Minh T, Tran-Manh C, Giessl A, Bergmann C, Gyorfi AH, et al. Impaired Mitochondrial Transcription Factor A Expression Promotes Mitochondrial Damage to Drive Fibroblast Activation and Fibrosis in Systemic Sclerosis. Arthritis Rheumatol. 2022;74(5):871-81.
